# Valorization of *Agave* Leaf Juice for Optimized *Kocuria sediminis* AS04 Production and Its Delivery via Immobilized Films to Mitigate Saline Stress in *Capsicum annuum* var. *glabriusculum*

**DOI:** 10.3390/biotech15030067

**Published:** 2026-08-15

**Authors:** Claudia Estefania Cabrera-Muro, Rosa María Camacho-Ruiz, Miguel Angel Lorenzo-Santiago, Jacobo Rodriguez-Campos, Silvia Maribel Contreras-Ramos

**Affiliations:** 1 Unidad de Tecnología Ambiental, Centro de Investigación y Asistencia en Tecnología y Diseño del Estado de Jalisco A.C. (CIATEJ), Normalistas No. 800, Colinas de la Normal, Guadalajara C.P. 44270, Jalisco, Mexico; clcabrera_al@ciatej.edu.mx; 2 Unidad de Biotecnología Industrial, Centro de Investigación y Asistencia en Tecnología y Diseño del Estado de Jalisco A.C. (CIATEJ), Camino Arenero No. 1227, El Bajío del Arenal, Zapopan C.P. 45019, Jalisco, Mexico; rcamacho@ciatej.mx; 3 Unidad de Servicios Analíticos y Metrológicos, Centro de Investigación y Asistencia en Tecnología y Diseño del Estado de Jalisco A.C. (CIATEJ), Normalistas No. 800, Colinas de la Normal, Guadalajara C.P. 44270, Jalisco, Mexico

**Keywords:** *Agave* waste, PGPB, *Kocuria sediminis*, pepper, saline soils

## Abstract

Halotolerant plant growth-promoting bacteria offer a sustainable strategy to improve crop performance under saline conditions. This study evaluated *Agave tequilana* Weber var. Azul leaf juice was used as an alternative growth medium for *Kocuria sediminis* AS04, and the bacterium’s ability to alleviate salt stress in chiltepin (*Capsicum annuum*) was evaluated. The chiltepin seedlings were grown in a specialized chamber and exposed to higher salt levels (200, 400, and 600 mM NaCl) for 10 days. During this time, the protective effect of *K. sediminis* AS04, which was held in a polymer film, was examined. *K. sediminis AS04* grew well in a medium containing 25% *agave* juice and urea, reaching a density of 1.3 × 10^10^ CFU mL^−1^. Compared with conventional Tryptic Soy Broth, the medium formulated from agave leaf juice and urea could reduce the cost per kilogram of biomass by approximately 4-fold. Under severe salinity stress, plants inoculated with immobilized *K. sediminis* at 600 mM NaCl exhibited the highest shoot biomass (0.31 g^−1^ plant), root length (50.7 mm), and proline accumulation (11.25 µmol g^−1^ fresh weight), whereas uninoculated plants displayed reduced biomass (0.16 g plant^−1^) and shorter roots (34.5 mm). At 600 mM NaCl, inoculation increased shoot biomass, root length, and plant survival by approximately 94%, 47%, and 200%, respectively, compared with uninoculated seedlings. This method values agro-industrial waste and enhances chiltepin performance under high-salinity conditions. It promotes sustainable production and helps create affordable inoculants for agricultural biotechnology.

## 1. Introduction

Soil salinity represents a critical threat to global agricultural productivity. Saline soil, characterized by electrical conductivity exceeding 4 dS m^−1^, affects 1381 million hectares of agricultural land (10.7% of the global surface), resulting in yield losses of up to 70% [1]. This phenomenon affects 33% of the world’s cultivated areas [2]. Saline compounds, such as sodium chloride (NaCl), generate reactive oxygen species (ROS) that induce osmotic and ionic stress, damaging cellular structures in plants and reducing photosynthetic rates and growth [3].

Salinity affects 60% of agricultural soils in Mexico [4], with irrigated agriculture experiencing a higher incidence of salinity buildup in dry areas, which make up over 50% of the country [5]. This affects important crops, both economically and culturally, such as chiltepin pepper *(Capsicum annuum* var. *glabriusculum*) [6]. This native product from northern Mexico has increased its popularity in the culinary market, positioning itself as one of the most in-demand chili peppers and commanding prices up to 40% higher than those of commercial pepper varieties [7].

National production, predominantly artisanal, is concentrated in Sonora, Sinaloa, Baja California Sur, and Chihuahua, with an estimated annual output of 5–10 tons of dried chiltepin, according to reports from producers in southern Sonora and Sinaloa [8]. Although the species exhibits remarkable genetic variability and resilience under adverse conditions, including tolerance to NaCl concentrations up to 300 mM in irrigation water [9], populations have declined considerably, driven by increasing demand and a lack of conservation strategies [8].

Salinity poses a significant risk to conservation and productive expansion in arid regions. To address these salt stress challenges, plant growth-promoting bacteria (PGPB) have emerged as a promising solution. PGPB helps plants grow by fixing atmospheric nitrogen, solubilizing phosphate, producing siderophores, and synthesizing plant hormones [10]. Additionally, these microorganisms enhance crop resilience by conferring stress tolerance through osmolyte production, regulation of ion homeostasis, and activation of antioxidant enzymes [10]. Such mechanisms translate into measurable improvements in biomass, root development, and proline accumulation, with consistent results reported across diverse crop species under saline stress [11].

Using PGPB strains has been shown to alleviate salt stress in *Capsicum* species by significantly enhancing the plants’ physical, biological, and chemical characteristics [12]. The genus *Kocuria* exhibits strong halotolerance (up to 10% NaCl) and produces multiple plant growth-promoting compounds, including indole-3-acetic acid, siderophores, and ammonium, while solubilizing phosphorus [13]. Application of *Kocuria* species to salt-stressed crops has improved germination and other growth parameters [14]. Nevertheless, the performance of *Kocuria* in *Capsicum annuum* var. *glabriusculum* exposed to salinity has not been evaluated.

In practice, PGPB formulations require cellular concentrations typically in the range of 1 × 10^7^ to 1 × 10^9^ CFU mL^−1^ or 1 × 10^7^ to 1 × 10^9^ CFU g^−1^ to ensure successful rhizosphere colonization [15]. However, achieving these densities requires high-cost culture media, such as tryptone-based broths (TSB), which account for 60–70% of total biomass production costs [16]. For *Kocuria*, a previous study optimized TSB-based cultivation, achieving a dry biomass yield of 13.3 g L^−1^ [17]. Considering the tryptone costs of 210 USD per kg [18], the estimated production cost is approximately 500 USD per kg of dry biomass. These high production costs create a significant economic barrier to large-scale agricultural implementation. An alternative approach to reducing the cost of bacterial biomass production is to use agricultural residues as components of culture media.

In 2024, the tequila industry processed 1.8 million tons of *agave* (CRT, 2025), generating approximately 720,000 tons of *agave* leaf residues, given that leaves account for approximately 40% of fresh *agave* weight [19]. Recent advances in microbial inoculant formulation emphasize that encapsulating PGPR in natural polysaccharide matrices and combining them with solid carriers, including agro-industrial residues and protective additives such as skim milk or sugars, can extend shelf life, improve stress tolerance of the inoculant, and enable a more controlled release of cells in soil, enhancing biofertilizer performance under field conditions [20]. *Agave* juice from leaf residues contains carbohydrates such as fructans, fructose, and glucose, which serve as carbon sources for microbial cultures, potentially offering a low-cost alternative to conventional media while reducing environmental impact through waste valorization [21]. Therefore, the present study aims to evaluate the effectiveness of *agave* (*Agave tequilana* Weber var. Azul) leaf juice as a cost-effective culture medium for *Kocuria sediminis* AS04 biomass production and, subsequently, to evaluate its effect on immobilizing bacteria to mitigate salt stress in *Capsicum annuum* var. *glabriusculum*. To our knowledge, the use of *A. tequilana* leaf juice as the main carbon source for biomass production of a halotolerant PGPB inoculant, specifically *K. sediminis* AS04, has not been previously reported.

## 2. Materials and Methods

**Figure 1:**
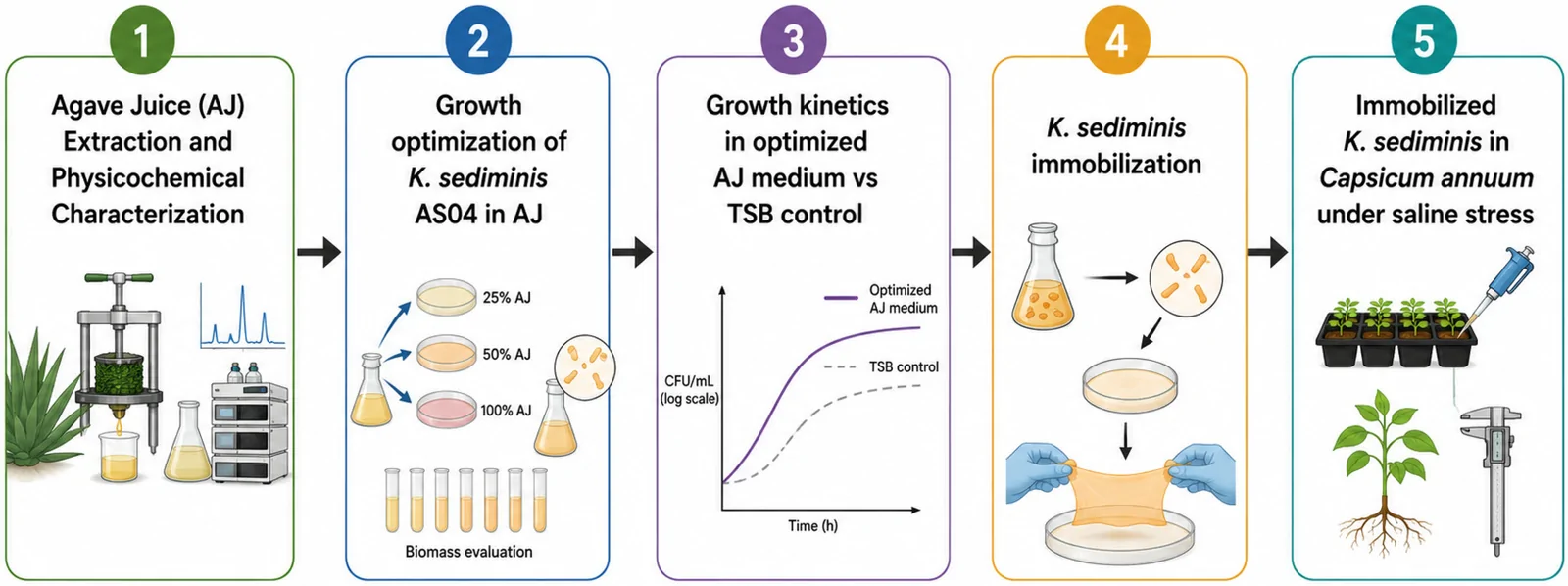
Schematic representation of the experimental workflow from *Agave* juice extraction to application of the immobilized *Kocuria sediminis* AS04 inoculant in *Capsicum annuum* under saline stress.

This study comprised five sequential stages: (1) *Agave* juice extraction and physicochemical characterization, (2) growth optimization of *Kocuria sediminis* AS04 in agave-based media, (3) comparison of growth kinetics in optimized agave juice versus TSB, (4) immobilization of *K. sediminis* in an agave-derived biopolymer film, and (5) application of the immobilized inoculant to *Capsicum annuum* under saline stress. These stages are summarized schematically in Figure 1.

### 2.1. Raw Material Collection and Agave Juice Extraction

*A. tequilana* leaf sections were collected from Cuquio, Jalisco, Mexico (21.03071° N, 103.04635° W). Leaf tissue was weighed before and after removal of the cuticles and upper epidermis, then fragmented into 7–10 cm pieces, washed with distilled water, and stored at 4 ± 2 °C in plastic bags until processing [22].

Juice extraction was performed using a CARVER hydraulic press (Carver Inc., Wabash, IN, USA) at 25 ± 2 °C, with up to 20 tons of pressure applied. The obtained juice was centrifuged at 4000 rpm for 10 min at 4 °C using a Gyrozen 1248R centrifuge (Gyrozen Co., Ltd., Daejeon, South Korea), filtered through 0.45 μm membranes, and stored at −80 ± 2 °C until use.

### 2.2. Characterization of Agave Juice

Physical parameters were determined using a HI 3512 multiparametric potentiometer (Hanna Instruments, Woonsocket, RI, USA) with specific electrodes for pH (HI 1131B with HI 7662-T probe) and electrical conductivity (HI 76310). Measurements were performed in triplicate at 25 ± 2 °C with a 2 min stabilization period, and soluble solids (°Brix) were quantified using a digital refractometer (Atago, Tokyo, Japan).

Total carbohydrates were determined by the colorimetric method of Dubois et al. [23], using a glucose calibration curve (10–80 mg L^−1^). Samples were analyzed in triplicate using a DR 2800 spectrophotometer (Hach Company, Loveland, CO, USA) at 490 nm.

Carbohydrate profile was characterized by HPLC-RID using an AMINEX HPX-87C column (Bio-Rad, Hercules, CA, USA, 300 × 7.8 mm). Samples were diluted 1:10, filtered (0.45 μm), and analyzed using ultrapure water as mobile phase (flow rate 0.6 mL min^−1^), 85 °C, injection volume 10.0 μL). Each sample was processed in triplicate with duplicate technical injections.

Total phenolic compounds were quantified by the Folin–Ciocalteu method [24] using gallic acid as a standard (100–1000 mg L^−1^). The reaction was developed for 2 h at 25 ± 2 °C, and absorbance was measured at 765 nm using a DR 2800 spectrophotometer.

Total nitrogen was determined by Hach method 10071 (alkaline digestion with persulfate, reading at 420 nm) and total phosphorus by Hach method 10127 (molybdovanadate with acid digestion, reading at 420 nm). Both determinations were performed using the Test ‘N Tube™ system following the manufacturer’s specifications.

Total organic carbon content was quantified using a TOC-L-CS analyzer (Shimadzu, Kioto, Japan) by high-temperature combustion with infrared detection, as specified in Mexican standard NMX-AA-187-SCFI-2021 [25]. *Agave* leaf juice was filtered through 0.22 μm membranes before analysis. All samples were diluted 1:100 with Milli-Q water to fall within the equipment detection range.

### 2.3. Bacterial Strain and Culture Conditions

*Kocuria sediminis AS04* was previously isolated from air samples collected in the metropolitan area of Guadalajara, Mexico, and identified using MALDI-TOF mass spectrometry and molecular sequencing [26].

### 2.4. Growth of K. Sediminis AS04 in Agave Juice

Growth of *Kocuria sediminis* AS04 in the presence of *agave* leaf juice was first evaluated on solid medium. Tryptic Soy Agar plates consisted of casein peptone (15 g L^−1^), soy peptone (5 g L^−1^), NaCl (5 g L^−1^) and agar (15 g L^−1^), which were supplemented with *agave* juice at 25%, 50%, and 100% (*v*/*v*), and a non-supplemented TSA plate served as the control. Bacterial growth was assessed using the drop plate (Miles and Misra) [27]. A bacterial suspension from a single colony was prepared in sterile saline solution (0.9% *w*/*v* NaCl) and serially diluted from 10^−1^ to 10^−6^. Three 10 μL drops from each dilution were placed onto separate sectors of each plate in triplicate for each treatment. Plates were incubated for 48 h at 34 ± 2 °C, within the 30–38 °C growth range previously reported for *K. sediminis* AS04 [17], and the presence or absence of growth at each dilution and treatment was recorded to assess the strain’s tolerance to the compounds present in the medium.

#### 2.4.1. Culture Media Preparation and Inoculum

Based on the growth limits of *K. sediminis AS04* established in solid medium, juice concentrations of 25, 35, and 45% (*v*/*v*) were selected for liquid cultures. Each medium contained *agave* leaf juice, tryptone (20 g L^−1^), and NaCl (5 g L^−1^) and was adjusted to a final volume with distilled water. The control Tryptic Soy Broth (TSB) medium contained only tryptone (20 g L^−1^) and NaCl (5 g L^−1^), without agave leaf juice. The pH of all media was adjusted to 7.0 ± 0.5, and the media were sterilized by autoclaving at 121 °C for 20 min at 15 psi before use. For inoculation, a pre-inoculum was prepared by cultivating *K. sediminis* AS04 in TSB for 48 h at 34 ± 2 °C with orbital shaking (130 rpm), conditions previously optimized [17]. From this culture, the inoculum was adjusted to an optical density of 0.1 at 600 nm using Equation (1).

(1) $$\begin{array}{cccc} & C_{1}\;V_{1}=C_{2}\;V_{2} & & \end{array}$$

where *C*1 is the initial concentration, *V*1 is the initial volume, *C*2 is the final concentration, and *V*2 is the final volume.

#### 2.4.2. Viable Cell Counts (CFU mL^−1^)

Viable cell counts were determined by serial dilution and the spread plate method. Cultures were serially diluted (10^−6^ to 10^−8^) in sterile saline (0.9% *w*/*v* NaCl), and 100 μL aliquots were plated on standard methods agar. Following incubation at 34 ± 2 °C and 48 h, colonies were counted on plates with 30–300 colonies. CFU mL^−1^ values were calculated using Equation (2).

(2) $$\begin{array}{cccc} & {\mathrm{CFU}\;(\mathrm{mL}}^{-1})=\frac{\mathrm{number}\;\mathrm{of}\;\mathrm{colonies}\times \mathrm{dilution}\;\mathrm{factor}}{plated\;volume\;(mL)} & & \end{array}$$

where the *dilution factor* is the inverse of the dilution, and the *plated volume* is 0.1 mL.

#### 2.4.3. Optical Density and Dry Biomass Determination

Optical density (OD) at 600 nm was measured on a DR 5000 UV─Vis spectrophotometer (Hach, Loveland, CO, USA), using uninoculated culture medium as the blank. For dry biomass determination, 10 mL of homogenized culture was filtered through a cellulose filter (0.45 μm) (Merck Millipore S-pak 47 mm, Darmstadt, Germany). The filters with retained biomass were dried at 105 ± 2 °C to a constant weight. Dry biomass concentration was calculated using Equation (3). After drying, the container’s combined weight and the dried sample were recorded as the *final weight*, while the weight of the filter paper was denoted as the *initial weight*. The differences between these values represent the dry biomass obtained from the 10 mL sample (*Volume of culture*).

(3) $$Dry\;weight\;\left({gL}^{-1}\right)=\frac{\left(\;Final\;weight-Initial\;weight\;\right)}{Volume\;of\;culture}$$

### 2.5. Evaluation of Nitrogen Sources on Kocuria Sediminis AS04 Growth

To formulate a cost-effective alternative to commercial Tryptic Soy Broth (TSB), an experiment was designed to replace its primary nitrogen source (tryptone). Once the optimal *agave* juice concentration was established, three economical nitrogen sources, such as ammonium nitrate, ammonium sulfate, and urea, were evaluated. Each source was added to the *agave*-based medium at a concentration of 2.6 g N L^−1^, equivalent to the nitrogen content in 20 g L^−1^ tryptone. The concentrations of *agave* juice and NaCl remained constant across all treatments. Two controls were included: an *agave*-based medium supplemented with 20 g L^−1^ of tryptone, and commercial TSB. Following incubation, bacterial growth was quantified by measuring optical density at 600 nm, viable cell count (CFU mL^−1^), and dry biomass (g L^−1^) following the same analytical procedures and calculations described before.

### 2.6. Growth Kinetics of K. sediminis

Based on the evaluation of *agave* juice concentrations and nitrogen sources, a culture medium was formulated for biomass production of *K. sediminis* AS04. Growth kinetics were evaluated in an *agave*-based medium and in Tryptic Soy Broth (TSB) used as a reference medium. The *agave*-based medium contained 25% (*v*/*v*) *A. tequilana* leaf juice, 5.8 g L^−1^ urea, 5 g L^−1^ NaCl, and distilled water, while TSB was prepared with 20 g L^−1^ tryptone and 5 g L^−1^ NaCl. The pH of the *agave*-based medium was adjusted to 7.0 ± 0.5, and both media were sterilized.

A pre-inoculum of *K. sediminis* AS04 grew in TSB to the exponential phase, and its optical density at 600 nm was measured to calculate the inoculum volume required to obtain an initial OD of 0.1. The calculated volume of inoculum was aseptically transferred into sterile 250 mL Erlenmeyer flasks containing 200 mL of either the proposed medium or the TSB reference medium, each prepared in triplicate. Cultures were incubated at 34 ± 2 °C and 130 rpm in an orbital shaker, conditions previously optimized for this strain, and samples were taken at 0, 4, 8, 12, 24, and 48 h post-inoculation to evaluate their growth.

At each sampling time, 20 mL of culture was aseptically withdrawn from each flask. Aliquots were used to measure optical density at 600 nm (using the corresponding uninoculated medium as blank), to determine viable cell counts and dry biomass (g L^−1^).

Maximum specific growth rate and doubling time were estimated from the exponential phase of the CFU-based growth curves in each medium using standard exponential growth equations [17]. These kinetic parameters were used to compare the growth behavior of *K. sediminis* AS04 in the *agave* juice-based medium and in the TSB reference medium. The maximum specific growth rate was calculated following Equation (4).

(4) $$\begin{array}{cccc} & \mu_{max}=\frac{lnX_{2}-lnX_{1}}{t_{2}-t_{1}} & & \end{array}$$

where $X_{1}$ and $X_{2}$ represent the viable cell concentrations (CFU mL^−1^) at times $t_{1}$ and $t_{2}$ (h) during the exponential phase, respectively. Subsequently, the doubling time was determined using Equation (5).

(5) $$\begin{array}{cccc} & DT=\frac{\ln2}{\mu_{\max}} & & \end{array}$$

### 2.7. Cellular Immobilization in Biopolymeric Formulation

Cell immobilization was employed to formulate *K. sediminis* AS04 into a biopolymeric film derived from fermented agave leaf juice, thereby confining the bacterium within a solid matrix during handling and storage. The liquid formulation tends to undergo desiccation when stored under semi-controlled conditions or when applied to seeds under uncontrolled environmental conditions after field applications, whereas a solid formulation that protects the bacterial cells could offer greater stability because desiccation is controlled during the process technology [28].

Compared to traditional solid carrier-based formulations, liquid formulations allow for better sterilization control and can maintain higher viable cell counts of beneficial microbes throughout the culture cycle, allowing for lower application rates.

Biomass production was carried out in the *agave*-based medium under the same incubation conditions described above, and viable cell counts were determined as previously described to confirm cell densities of 1.08 × 10^10^ CFU mL^−1^. The bacterial culture was transferred to conical tubes and centrifuged at 4000 rpm for 10 min. Supernatants were discarded under aseptic conditions, and the bacterial biomass was recovered for immobilization.

Bacterial immobilization was performed using a previously developed method [29]. Briefly, a biopolymer solution was prepared and homogenized under magnetic stirring until uniform. A control solution with identical composition but without bacterial cells was prepared in parallel. The mixture was poured into sterile Petri dishes at 20 mL per plate using the casting technique. Plates were dried under aseptic conditions in a Class II biosafety cabinet with continuous HEPA-filtered laminar airflow (Esco Lifesciences Group, Singapore) at 0.39 m s^−1^ and 25 ± 2 °C for 48 h until film formation. Dried films were carefully detached from the plates. The film also exhibited a water solubility of 97.66 ± 0.22% after 2.5 h of immersion, determined gravimetrically as described in [29]. Shelf life and stability of the immobilized cells were evaluated by storing the films at 35 ± 2 °C and determining viable cell counts at 0, 3, 6 and 9 weeks, following accelerated storage principles described in the CIPAC method MT 46.4 for pesticide formulations [30]. The solubility of the films was determined as described by Lorenzo-Santiago et al. [29].

### 2.8. Effect of K. Sediminis Immobilized in Plants of Capsicum annuum var. glabriusculum Under Saline Stress

Forty-day-old seedlings of chiltepin pepper (*C. annuum* var. *glabriusculum)* obtained from a commercial greenhouse were transplanted individually into 148 mL polyethylene terephthalate (PET) pots containing 15 g of sterilized substrate mixture (40% soil, 40% coconut fiber, 20% perlite), irrigated with 10 mL of sterilized water, and acclimated for 24 h at room temperature. Uniform, healthy seedlings were selected and transferred to a growth chamber (16 h light/8 h dark photoperiod, 28 ± 2 °C, 65–70% relative humidity), where they received 5 mL of sterile water per plant for an additional 24 h of acclimatization.

Stress was induced by irrigating plants with NaCl solutions at 200, 400, or 600 mM prepared from reagent-grade NaCl in sterile distilled water. Each plant received 5 mL daily of the corresponding solution or sterile water for 10 consecutive days to promote gradual salt accumulation while avoiding substrate leaching. These NaCl concentrations were selected based on a previous study in chiltepin pepper, in which seedlings irrigated with 300 mM NaCl maintained relative growth comparable to the non-saline control, indicating halotolerant behavior [9]. 200, 400, and 600 mM used to generate a gradient from moderate to severe salinity stress.

The experiment followed a completely randomized design with nine treatments and ten replicates per treatment (90 plants), allowing paired comparisons between inoculated and non-inoculated plants at each level. Additionally, three non-saline controls were included to evaluate normal plant growth, the independent growth-promoting effect of *K. sediminis* AS04, and the effect of the biopolymeric matrix. The detailed description and coding of each experimental treatment are summarized in Table 1. Chiltepin plants (*C. annuum* var. *glabriusculum*) exhibited uniform growth at the start of the experiment, with similar sizes and leaf numbers across all treatments.

In treatments receiving biopolymeric films with *K. sediminis* AS04, the immobilized formulation was applied as an aqueous suspension prepared by dissolving 0.5 g of film in 250 mL of sterile water under aseptic conditions, after cutting the dried films with a sterile scalpel and handling them with sterile forceps in a Class II biosafety cabinet, with constant agitation until fully dissolved, yielding a concentration of 2 mg mL^−1^. Each plant received a single 6.25 mL dose of this suspension, corresponding to 0.0125 g (12.5 mg) of biopolymeric film with immobilized *K. sediminis* AS04 per plant, applied on day 0 immediately after the first irrigation under the corresponding salinity treatment. The biopolymeric film maintained a viable cell count of 1.17 × 10^10^ CFU mL^−1^ in this solution, as determined by viable plate cell counting as described in Section 2.4.2. The application rate was calculated based on an average chiltepin density of 16,000 plants per hectare and a dose of 200 g ha^−1^ of the bacterial-immobilized matrix. In the matrix control treatment, the same volume and amount of film were applied using bacteria-free film. At the end of the 10-day treatment period, substrate electrical conductivity was monitored using the saturated paste method with a multiparameter meter (HI 3512, Hanna Instruments) to verify the proper induction of the salinity stress [31].

### 2.9. Plant Growth and Physiological Measurements

Plants were inspected for symptoms of salt stress, including wilting, leaf chlorosis, and leaf abscission, and survival was recorded. At the end of the experimental period, plants were removed from the substrate, gently washed to remove adhering particles, blotted on absorbent paper, and stem length, stem diameter, root length and root thickness (overall width of the root system) were measured using a digital electronic caliper (DEC, Prodical 1101, Accurex, Loveland, CO, USA).

For dry biomass determination, seven plants per treatment were dried individually in paper bags at 80 ± 2 °C for 4 days in a forced-air oven (BINDER, FD23-20L, Tuttlingen, Germany), transferred to a desiccator for 30 min, and weighed on an analytical balance to determine dry biomass per plant.

#### 2.9.1. Proline Determination

Free proline content was determined by the colorimetric method described by Bates [32]. All leaves per plant were cut, and a sample of 0.5 g fresh weight FW was homogenized in a mortar with 10 mL of 3% (*w*/*v*) sulfosalicylic acid, and the homogenate was centrifuged at 4000 rpm for 10 min; the supernatant was used for the colorimetric reaction. For color development, 2 mL of extract was mixed with 2 mL of acid ninhydrin reagent and 2 mL of glacial acetic acid in test tubes, incubated at 100 ± 2 °C for 60 min in a water bath, and then rapidly cooled on ice. After incubation, 5 mL of toluene was added, the tubes were vigorously shaken for 15 s and left in the dark at room temperature for at least 20 min to allow complete phase separation; the upper toluene phase, showing a brick-red color, was collected, and its absorbance was read at 520 nm using a reagent blank without plant extract. Proline concentration was calculated from a standard curve prepared with L-proline (0–80 µg mL^−1^) and expressed as millimoles of proline per gram of fresh weight (µmol g^−1^ FW).

#### 2.9.2. Glycine Betaine Determination

Glycine betaine (GB) content was determined in dried leaf tissue using a colorimetric method based on the formation of a periodide complex, as described by Grieve and Grattan [33], with minor modifications. Plant material was dried at 80 ± 2 °C for 4 days, finely ground, and 0.50 g of dry tissue was extracted with 20 mL of deionized water under mechanical shaking for 24 h at 25 ± 2 °C; the extracts were filtered and diluted 1:1 with 2 N sulfuric acid. Aliquots of 0.5 mL were transferred to thick-walled glass tubes pre-chilled on ice for 1 h, and 0.20 mL of cold KI-I_2_ reagent was added; the mixtures were vortexed and kept at 0–4 °C for 16 h to allow formation of the GB-periodide complex. Samples were then centrifuged at 10,000 rpm for 15 min at 0 ± 2 °C. The supernatant was carefully discarded, and the crystalline periodide complex was dissolved in 9.0 mL of 1,2-dichloroethane by vigorous vortexing. After 2 h, the absorbance of the organic phase was measured at 350 nm using a reagent blank without plant extract. GB concentration was calculated from a standard curve prepared with glycine betaine solutions (500–200 µg mL^−1^) subjected to the same reaction procedure, and results were expressed as µmol glycine betaine per g dry weight (µmol g^−1^ DW).

### 2.10. Statistical Analysis

For each response variable, the mean and standard deviation were calculated. Data were checked for normality and homogeneity of variances using the Shapiro–Wilk and Levene tests, respectively. When these assumptions were met, differences among treatments were evaluated by a one-way ANOVA followed by Tukey’s multiple comparison test (*p* < 0.05). Otherwise, the nonparametric Kruskal–Wallis test was applied. No data transformation procedures were used; instead, nonparametric tests were preferred when the assumptions of ANOVA were violated. For pairwise comparisons between two independent groups, Welch’s *t*-test was used (*p* < 0.05). These analyses were performed using Minitab (version 19; Minitab LLC, State College, PA, USA). Additionally, to evaluate the overall relationships between growth and physiological parameters across all treatments, a Principal Component Analysis (PCA) was conducted in R (version 4.5.2; R Core Team, 2025) using RStudio. The PCA was performed using the measured physiological and biochemical variables such as root thickness (overall width of the root system), root length, dry biomass, stem thickness, stem length, proline and glycine betaine content. Only samples with complete datasets were included in the analysis. The dataset consisted of 10 replicates per treatment, although treatments with reduced plant survival contributed fewer complete observations. No missing values were imputed. Prior to PCA, all variables were mean-centered and standardized to eliminate the influence of differences in measurement scales.

## 3. Results and Discussion

### 3.1. Characterization of Agave Juice

Processing 14.8 kg of whole *A. tequilana* Weber leaves resulted in 8.76 kg of usable pulp (59.2%) after removal of spines and cuticles. Mechanical extraction of this pulp yielded 2.02 L of juice, corresponding to 13.6% of the initial leaf weight and 23.1% of the processed pulp. The extracted juice was a greenish-brown liquid with visible fibrillar material and a high soluble solids content of 44 ± 1.0 °Brix, which was above the typical range of 23.68–30.70 °Brix reported for *agave* leaf juice [34]. This indicated that the leaf juice is particularly concentrated in soluble solids, likely due to its high sugar and fructan content. Physicochemical characterization (Table 2) revealed an acidic pH of 5.14, comparable to that reported for *A. salmiana* (5.39 ± 0.56) and *Agave durangensis* (5.29 ± 0.05) [35], and consistent with the moderate acidity characteristic of *agave* tissues rich in fructans.

The total inulin content reached 47.9 g L^−1^, which falls slightly above the 38.41–43.86 g L^−1^, range reported for aqueous extracts of *Agave americana* [39], indicating that the leaf juice constitutes an important source of fructans with potential use as a carbon source. The total organic carbon (TOC) content of the leaf juice reached 27.44 g L^−1^, which is comparable to the 32.03 g L^−1^ reported for agave IAC4 juice [41]. The phenol content (1.27 g GAE L^−1^) was higher than the 0.48 g GAE L^−1^ reported for aguamiel from *Agave salmiana* and *Agave mapisaga* [43], but lower than processed aqueous extracts from *A. salmiana* leaves (2.91 g GAE L^−1^) [40]. The total phosphorus content in the leaf juice was 1.21 g L^−1^, notably higher than the 0.087 g L^−1^ reported for *Agave* sp. *IAC4* juice [41], indicating that the juice naturally satisfies the phosphorus requirements for microbial development. In contrast, total nitrogen in the extracted juice was 0.22 g L^−1^, higher than the 0.087–0.095 g L^−1^ reported for *Agave tequilana* must [42], thereby justifying nitrogen supplementation in the formulated culture medium. These variations in macronutrient proportions in *agave* leaves are strongly influenced by factors like soil fertilization levels, crop age, and prior nutrient assimilation by native endophytic bacteria [34].

*Agave* juice can be viewed as an agro-industrial residue, like other low-cost substrates used for bacterial biomass production. Soybean molasses, for example, have been optimized as a carbon source for rhizobia inoculants [44]. Molasses combined with corn steep liquor has likewise been used as an inexpensive carbon–nitrogen source pair to obtain high biomass yields of the PGPR *Rhodopseudomonas palustris* PS3 [45], supporting the use of agave juice as a sugar-rich substrate for PGPB growth.

### 3.2. Growth of K. sediminis AS04 in Agave Juice

**Figure 2:**
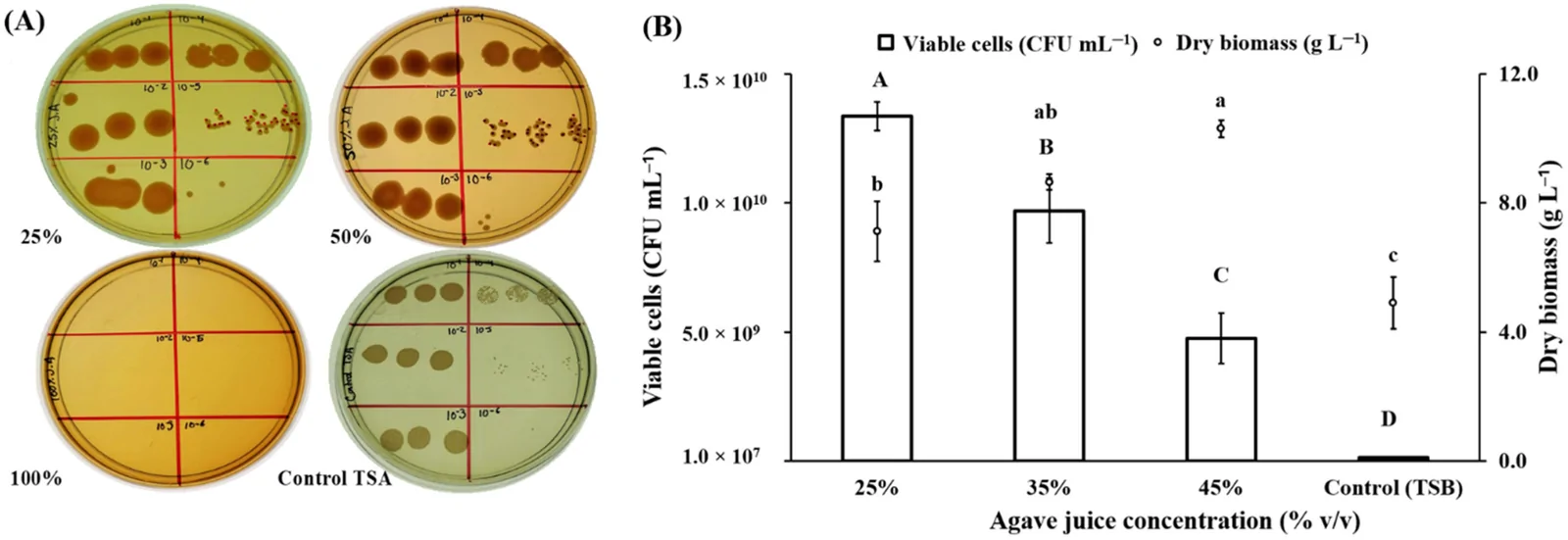
Growth of *K. sediminis* in *agave* juice medium. (**A**) Representative agar plates showing viable-cell growth at different Agave juice concentrations (25%, 50%, and 100%) and in the control medium (TSA)., and (**B**) Viable cell counts (bars, left *y*-axis; CFU mL^−1^) and dry biomass (circles, right *y*-axis; g L^−1^) obtained after cultivation in media containing 25, 35, and 45% (*v*/*v*) agave leaf juice or the control medium (TSB). Bars and points represent mean ± standard deviation (*n* = 3). Capital letters: significant differences in viable cells; lowercase letters: significant differences in dry biomass (Tukey’s test, *p* < 0.05).

*Kocuria sediminis* AS04 reached population densities of 1.06 × 10^8^ CFU mL^−1^ on the commercial TSA control after 48 h of incubation (Figure 2A). Growth on media with 25% *A. tequilana* leaf juice achieved a nominal increase of 12.2% in bacterial yield, reaching 1.2 × 10^8^ CFU mL^−1^, while the 50% juice treatment yielded 1.07 × 10^8^ CFU mL^−1^. At these concentrations, the juice provides 6.20 g L^−1^ and 12.4 g L^−1^ of total carbohydrates, respectively. Both juice concentrations proved statistically comparable to commercial control. In contrast, no bacterial growth was detected at the 100% juice concentration (24.8 g L^−1^). The total carbohydrate content associated with each juice concentration is detailed in Table 3.

After 48 h, all *agave* juice treatments significantly outperformed TSB controls. Regarding viable counts, the 25% concentration yielded the highest values (1.34 × 10^10^ CFU mL^−1^), approximately 88-fold above TSB levels (1.52 × 10^8^ CFU mL^−1^), followed by 35% (9.70 × 10^9^ CFU mL^−1^) and 45% (4.77 × 10^9^ CFU mL^−1^) (Figure 2B), indicating that increasing juice concentration beyond 25% progressively reduced cell density despite the higher sugar content (Figure 2B). A similar trend was observed for optical density, with values decreasing from 1.37 ± 0.015 at 25%, to 1.17 ± 0.05 at 35%, and 0.88 ± 0.09 at 45% (Supplementary Material Figure S1).

Dry biomass production showed the opposite trend to cell density, reaching a significant maximum at 45% juice (10.30 ± 0.27 g L^−1^), more than double the yield of the commercial TSB control (4.91 ± 0.80 g L^−1^) (Figure 2B).

The robust growth of *K. sediminis* AS04 at 25% and 50% *agave* juice indicates that endogenous carbohydrates serve as an effective carbon source. In contrast, complete inhibition at 100% juice (24.8 g L^−1^ total carbohydrates) is consistent with osmotic stress induced by the high concentration of soluble sugars (Figure 2A), which raises medium osmolarity, promotes water loss from the cells, and leads to cytoplasmic dehydration, disrupting cell turgor and other physical properties that support bacterial growth [46]. Under such high-osmolarity conditions, bacteria rely on the synthesis and uptake of compatible solutes via tightly regulated, energy-demanding systems to restore cytoplasmic hydration and turgor, and this adaptive response incurs a metabolic cost that can limit nutrient acquisition and cell division when the osmotic load is extreme [47]. This interpretation is consistent with the concentration-dependent antimicrobial effect of sugar solutions described in sucrose-rich media [48]. For *K. sediminis* AS04, the inhibitory threshold is between 12.4 and 24.8 g L^−1^ of total carbohydrates, reflecting a higher osmotic sensitivity compared to other bacterial models such as *Escherichia coli*, *Pseudomonas* spp., *Klebsiella* spp., and *Staphylococcus aureus*, which require sucrose concentrations of 7 to 18.6 g L^−1^ to show comparable inhibition [49]. This osmotic pattern was further confirmed in a liquid medium, where viable counts decreased as juice concentration increased (Figure 2B).

The inverse relationship between dry biomass and cell density at higher juice concentrations is consistent with hyperosmotic stress responses, where *E. coli* exposed to high solute concentrations undergoes intracellular water loss, cell volume reduction, and prolonged cell cycles, resulting in cells with higher dry mass density and DNA content per unit volume [50]. Osmotic stress can also promote cytoplasmic condensation, increasing internal density without compromising total biomass [51]. These mechanisms could explain why elevated juice concentrations in *K. sediminis* AS04 slowed cell division while favoring the synthesis of structural or reserve components, producing fewer but heavier cells. Furthermore, *agave*-derived compounds have been shown to modulate microbial biomass architecture by inducing structural reorganization and enhancing exopolysaccharide production [52].

Optical density declined less sharply than viable counts as *agave* juice concentration increased (Figure S1, Supplementary Material). While OD decreased gradually, CFU counts dropped by almost two orders of magnitude, indicating that cells growing in concentrated juice can generate comparable turbidity with fewer viable units. This behavior is consistent with the fact that OD reflects not only cell number but also cell size and shape [53]. In *Kocuria*, carotenoid synthesis, including β-cryptoxanthin and rhodovibrin, serves as a protective response to stress [17,54]. Carotenoid absorption in the visible region occurs at approximately 400–550 nm [55]. The pigmentation of the cells may have contributed to light scattering, thereby partially influencing turbidity readings independently of cell numbers.

Lower juice concentrations support higher viable cell counts with minimal variability, making the 25% treatment optimal for inoculum production. *Agave* juice consistently exceeded TSB productivity across all concentrations. Additionally, replacing 25% of the water in the fermentation medium with *agave* juice could reduce water consumption by 25%, translating into significant economic and environmental benefits at an industrial scale.

### 3.3. Effect of Nitrogen Sources on K. sediminis AS04 Growth

**Figure 3:**
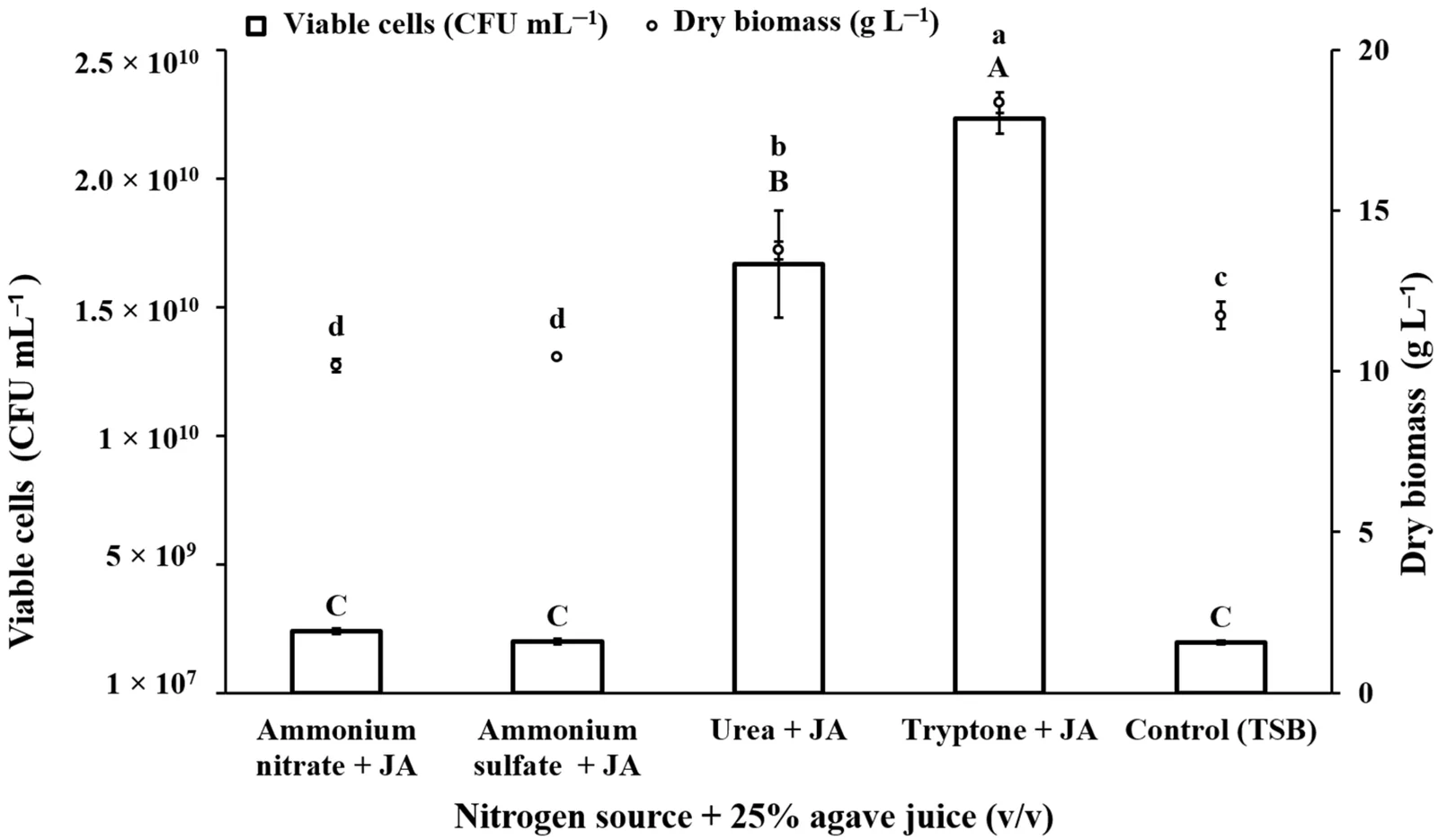
Effect of different nitrogen sources on *K. sediminis* AS04 growth in liquid medium supplemented with 25% (*v*/*v*) *A. tequilana* leaf juice after 48 h of incubation. Viable cell counts (bars, left *y*-axis; CFU mL^−1^) and dry biomass (circles, right *y*-axis; g L^−1^) were determined after cultivation in media supplemented with ammonium nitrate, ammonium sulfate, urea, or tryptone. TSB was included as the control medium. Bars represent viable cell counts (CFU mL^−1^), and circles represent dry biomass production (g L^−1^). Error bars denote the standard deviation of the mean (*n* = 3). Capital letters indicate statistically significant differences among treatments; uppercase for viable cells, lowercase for dry biomass (Kruskal–Wallis/Dunn’s test, *p <* 0.05).

Based on the superior growth observed at 25% *agave* juice, this concentration was selected to evaluate different nitrogen sources. *K. sediminis* AS04 growth varied depending on the nitrogen source provided. Cell viability and biomass production followed similar trends (Figure 3).

Tryptone and urea yielded significantly higher viable counts (2.23 × 10^10^ CFU mL^−1^) and dry biomass (18.36 g L^−1^), representing 11.3-fold and 56% increases over TSB, respectively. Urea also supported robust growth (1.67 × 10^10^ CFU mL^−1^; 13.76 g L^−1^), with viable counts 8.5-fold higher and biomass 17% greater than in TSB. In contrast, ammonium-based nitrogen sources yielded viability and biomass levels comparable to those in TSB. Tryptone supported the highest optical density (1.48 ± 0.01), followed by urea (1.35 ± 0.01). In contrast, inorganic sources such as ammonium nitrate (1.10 ± 0.02) and ammonium sulfate (0.74 ± 0.01) showed lower performance, comparable to or below that of TSB controls (0.50 ± 0.02) (Figure S2, Supplementary Material).

In the medium containing 25% *agave* juice, the nitrogen source significantly influenced the performance of *K. sediminis* AS04. The fact that tryptone consistently supported superior growth compared to TSB across all evaluated parameters suggests that tryptone derived from casein hydrolysis provides a more favorable nitrogen source for *K. sediminis* in *agave*-based media, consistent with similar responses in *Bacillus clausii*, where casein-derived tryptone promoted greater growth and metabolic activity than other nitrogen sources, attributed to its nutrient-rich amino acid profile [56].

Urea supported robust growth, reaching approximately 75% of tryptone’s performance, indicating that *K. sediminis* can effectively assimilate urea-derived nitrogen to support both cell replication and biomass accumulation. In contrast to ammonium-based sources, which tend to acidify the culture medium during assimilation, urea maintains a more stable pH, reducing the need for pH correction [57]. Compared to TSB, urea may offer a viable alternative for *K. sediminis* cultivation, with performance that is sufficiently competitive despite a slightly lower absolute yield than tryptone.

Both ammonium nitrate and ammonium sulfate supported viable cell counts only slightly above TSB levels, approximately 10-fold lower than with tryptone and 7–8-fold lower than with urea, suggesting that *K. sediminis* assimilates these nitrogen sources less efficiently under the present conditions. This behavior may indicate that ammonium-based nitrogen is less compatible with the nutritional demands of *K. sediminis* in this medium.

Beyond biological performance, economic considerations are equally critical for selecting a nitrogen source at an industrial scale. While tryptone supports superior growth and biomass production, its high cost, associated with casein-derived peptones, makes it impractical for large-scale biomass production. Urea at 5.6 g L^−1^ delivers an equivalent nitrogen input to 20 g L^−1^ tryptone, both providing 2.6 g N L^−1^, while dramatically reducing nitrogen-related costs. Based on current market prices (500 USD kg^−1^ for tryptone and 192 USD kg^−1^ for urea, both at cell culture grade; Merck) and normalizing to dry biomass yield obtained experimentally (18.36 g L^−1^ for tryptone and 13.76 g L^−1^ for urea), the estimated cost of producing 1 kg of dry biomass is approximately 545 USD with tryptone versus 78 USD with urea, representing a roughly 7-fold reduction. This cost advantage positions urea as an economically viable and operationally attractive nitrogen source for large-volume production of *Kocuria sediminis* AS04 inoculum intended for agricultural use, since the slight reduction in biomass yield relative to tryptone is far outweighed by its substantial economic benefits.

### 3.4. Growth Kinetics of K. sediminis AS04

**Figure 4:**
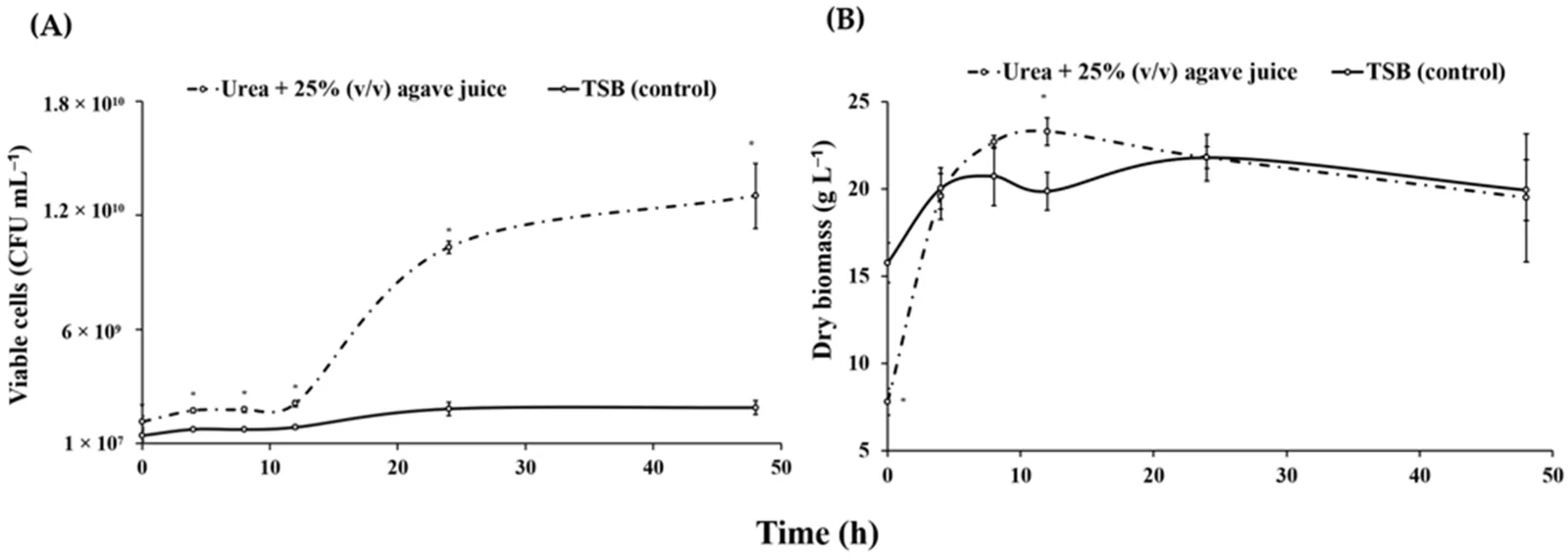
Growth kinetics of *Kocuria sediminis* AS04 over a 48 h incubation period comparing the proposed alternative medium (Urea + 25% *v*/*v agave* juice) and the commercial control (TSB). (**A**) Viable cell counts expressed in CFU mL^−1^. (**B**) Dry biomass production expressed in g L^−1^. Error bars represent the standard deviation of three biological replicates (*n* = 3). Asterisks (*) indicate statistically significant differences between the two media at a specific time point according to Welch’s *t*-test (*p* < 0.05).

Growth of *K. sediminis* AS04 was evaluated to be over 48 h in *agave*-urea medium and TSB by monitoring viable cell counts and dry biomass production. *K. sediminis* AS04 showed robust growth in both media, with the *agave*-urea medium supporting significantly higher cell densities compared to the TSB control consistently across all time points evaluated (*p* < 0.05, Welch’s *t*-test) (Figure 4A). In this medium, viable cells increased from 1.15 × 10^9^ CFU mL^−1^ to 1.30 × 10^10^ CFU mL^−1^ over 48 h. Growth followed a typical pattern, with an initial lag phase during the first 8 h, rapid exponential growth between 12 and 24 h, and stabilization at the stationary phase by 48 h. TSB, in contrast, supported more limited growth, with cell counts increasing from 4.30 × 10^8^ CFU mL^−1^ to 1.90 × 10^9^ CFU mL^−1^. The final cell density in the agave-urea medium was approximately 7-fold higher than in TSB, confirming the effectiveness of *agave* juice as an alternative culture medium for *K. sediminis* AS04. The agave-urea medium even surpassed the 5.7 × 10^9^ CFU mL^−1^ reported for *K. sediminis* AS04 in TSB under optimized incubation conditions [17], suggesting that *agave*-derived fructans and sugars present in *Agave* juice constitute a remarkably efficient carbon source for this strain.

Dry biomass accumulation showed a progressive increase in the urea + 25% *agave* juice medium from 7.8 ± 0.8 g L^−1^ to a maximum of 23.3 ± 0.8 g L^−1^ in the interval from 0 h to 12 h, followed by a slight decline to 19.5 ± 3.7 g L^−1^ by 48 h (Figure 4B). In contrast, TSB exhibited stable biomass production throughout the incubation, starting at 15.8 ± 1.1 g L^−1^ and remaining between 19.9 and 21.8 g L^−1^ from 4 to 48 h. Although the urea + 25% *agave* juice medium demonstrated a more dynamic initial growth phase, statistical analysis revealed no significant differences (*p* > 0.05, Welch’s *t*-test) in dry biomass yields between the two media during the stationary phase, indicating that both formulations support equivalent maximum biomass yields. These values exceed the 13.3 g L^−1^ reported for *K. sediminis* AS04 in TSB under optimized incubation conditions. [17].

When cell density and dry biomass are considered together, the *agave*-urea medium supported considerably higher cell populations while yielding equivalent total biomass to TSB, implying a lower dry matter content per cell. Bacterial dry matter content varies with nutrient availability and growth conditions [58], consistent with a medium that favors cell division over per-cell biomass accumulation.

*K. sediminis* AS04 exhibited faster exponential growth in the *agave*-urea medium with a μmax of 0.132 h^−1^ and a doubling time of 5.2 h. These values remain below those reported for the same strain in TSB under optimized incubation conditions (μmax of 0.184 h^−1^ and doubling time of 3.77 h) [17]. The *agave*-urea medium has not yet been systematically optimized, which could further improve its kinetic performance. Notably, despite this, viable cell counts and dry biomass production exceeded those previously reported for this strain.

### 3.5. Economic Implications of Using Agave Leaf Juice as a Substrate for K. sediminis

Production-cost analyses of microbial inoculants have reported values on the order of 100 USD kg^−1^ for powdered spore-based formulations and about 2.5 USD L^−1^ for liquid inoculants, when produced with conventional media and processes [59]. By contrast, low-cost agro-industrial substrates such as soybean molasses have reduced medium costs by about 70% while supporting high biomass yields in rhizobia inoculant production [44], and molasses combined with corn steep liquor has been used as an inexpensive carbon–nitrogen source pair to obtain high biomass yields of the PGPR *Rhodopseudomonas palustris* PS3 [45]. In this study, replacing Tryptic Soy Broth with the agave juice–based medium reduced medium cost per kilogram of biomass from approximately 300 to 70 USD kg^−1^, corresponding to a 77% cost reduction, making biomass production about four times cheaper and supporting agave juice as a competitive low-cost substrate for microbial inoculant production.

### 3.6. Cellular Immobilization in Biopolymeric Formulation

Under accelerated storage at 35 ± 2 °C, viable cell counts of immobilized cells decreased progressively over the 9 weeks. Counts dropped from 3.40 × 10^10^ CFU mL^−1^ at week 1 to 1.00 × 10^10^ CFU mL^−1^ at week 9, corresponding to a reduction of approximately 70%, with statistically significant differences between early and late sampling times. Film water solubility remained high throughout storage, consistently above 95%, with only minor variations of about 2 percentage points across weeks. These stability data are presented in Supplementary Material (Figure S3).

Accelerated stability tests have also been applied to starch-based films to estimate shelf life under ambient conditions, as shown for hydrolyzed corn starch packaging films [60]. Under accelerated storage at 35 ± 2 °C, the biopolymeric formulation showed a reduction in viable cells but still maintained final counts in the order of 10^10^ CFU mL^−1^, which remain above the typical minimum range (10^7^–10^9^ CFU mL^−1^ or CFU g^−1^) considered adequate for PGPB-based biofertilizer formulations [15]. This preservation of high viable cell densities, with film solubility consistently above 95%, suggests that the films can dissolve and release an inoculum suitable for practical application, including delivery via irrigation systems. The use of agave juice as a fermentation medium may provide fructans and inulin-type carbohydrates that act as protective carbon sources during storage, consistent with reports that residues and additives, such as sugars, can extend shelf life and improve the stress tolerance of microbial inoculants [20].

### 3.7. Effect of K. sediminis in Plants of Capsicum annuum var. glabriusculum Under Saline Stress

**Figure 5:**
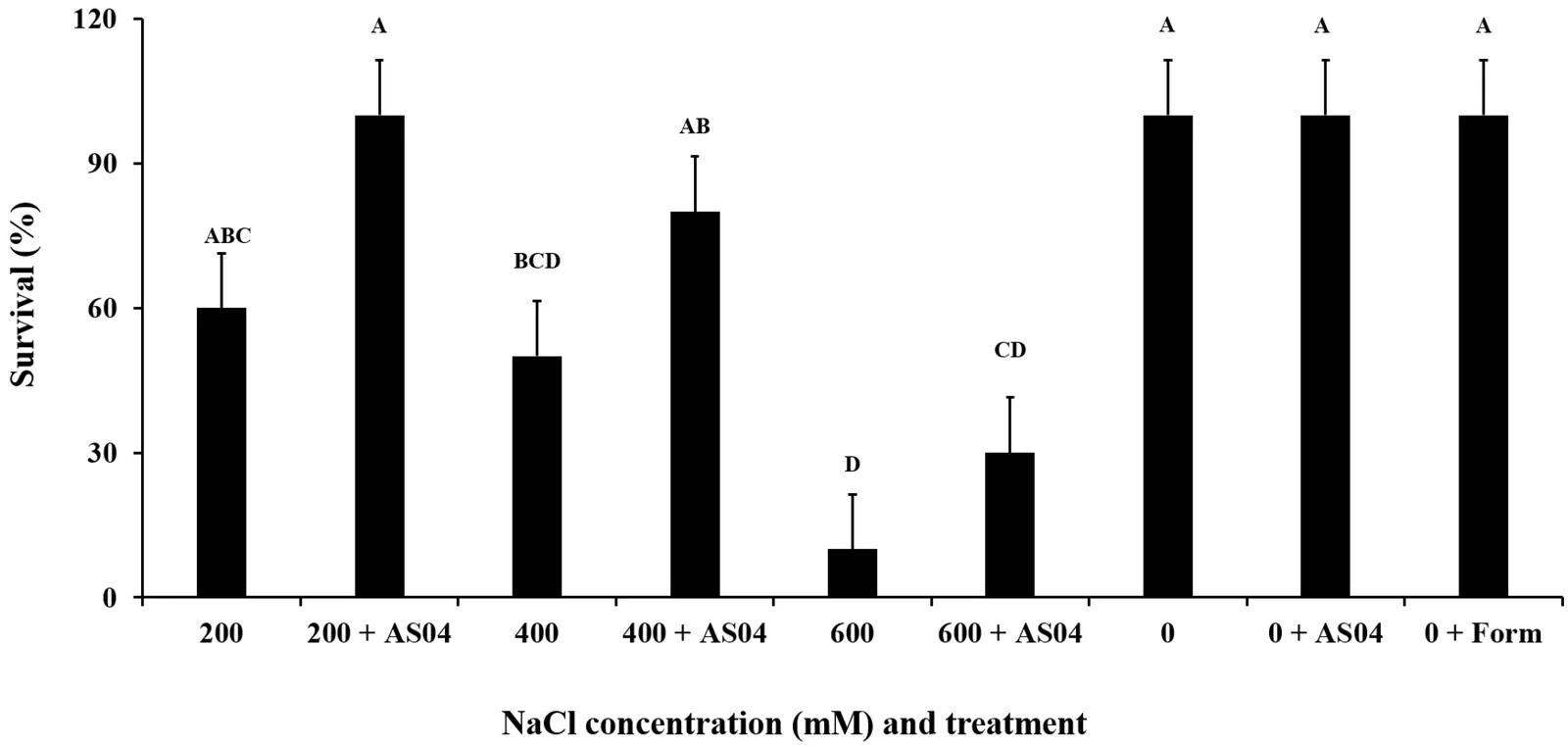
Effect of *K. sediminis* AS04 on the survival of plants after 10 days of saline stress (*n* = 10 plants per treatment). Plant survival (%) was evaluated under 0, 200, 400, and 600 mM NaCl in the absence or presence of *K. sediminis* AS04. Plants treated with the formulation (Form) under non-saline conditions were included as additional control. Data is expressed as percentage survival with 95% confidence intervals. Different letters above the bars indicate statistically significant differences among treatments according to one-way ANOVA followed by Tukey’s post hoc test (*p <* 0.05).

Plant survival decreased progressively with salinity in non-inoculated treatments, reaching 60% at 200 mM, 50% at 400 mM, and 10% at 600 mM (Figure 5) Inoculation with *K. sediminis* AS04 improved survival across all salinity levels evaluated, from 60% to 100% at 200 mM, from 50% to 80% at 400 mM, and from 10% to 30% at 600 mM (Figure 5). These differences in survival among treatments were statistically significant, supporting a protective effect of the bacterium under saline stress. All control treatments at 0 mM NaCl showed 100% survival.

**Figure 6:**
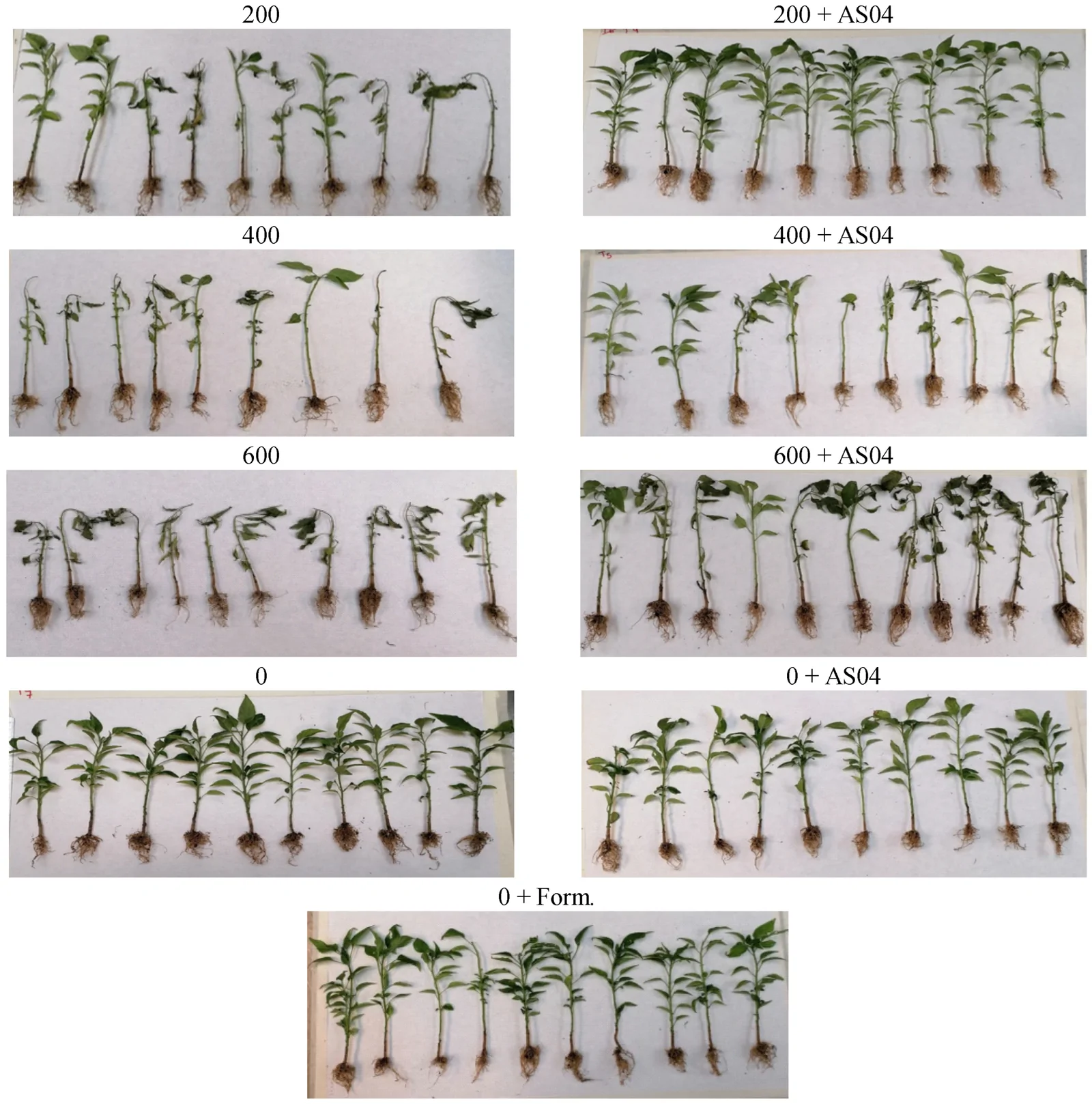
Representative photographs of *Capsicum annuum* var. *glabriusculum* plants after exposure to different salinity treatments and inoculation with *K. sediminis* AS04. Treatments are arranged from left to right and top to bottom as follows: 200 mM NaCl, 200 mM NaCl + AS04, 400 mM NaCl, 400 mM NaCl + AS04, 600 mM NaCl, 600 mM NaCl + AS04, 0 mM NaCl (control), 0 mM NaCl + AS04, and 0 mM NaCl + immobilized bacterial formulation (Form). The images show representative differences in plant morphology and vigor at the end of the experimental period.

After 10 days of salt stress, non-inoculated plants exhibited increasingly severe symptoms as NaCl concentration increased, including stunted growth, chlorosis, and necrosis, which were most pronounced at 600 mM. In contrast, plants treated with *K. sediminis* AS04 exhibited better growth and maintained greener foliage at all salinity levels. These observations were consistent with the substrate electrical conductivity rising from around 5 dS m^−1^ at 200 mM to about 7–8 dS m^−1^ at 600 mM in the substrate and remaining below 0.4 dS m^−1^ in 0 mM treatments (Figure 6 and Figure S5, Supplementary Material).

This progressive and significant decline in survival is consistent with the genus’s well-documented sensitivity to salinity. High mortality has been reported in *C. annuum* genotypes exposed to 3750 ppm (64.2 mM) and 7500 ppm (128.3 mM) NaCl before fruiting, with salinity inhibiting growth and yield by up to 80% compared to non-saline controls [61]. The symptoms observed in the present study correspond to the classic manifestations of ionic and osmotic stress that are particularly severe during juvenile stages, when osmotic adjustment mechanisms are not yet fully developed [62].

Beyond improving survival rates, the inoculation with *K. sediminis* AS04 preserved the overall plant morphology and root architecture under salt stress. Inoculated plants maintained a larger size and developed a more robust root system, contrasting with the stunted roots and reduced biomass of the non-inoculated plants (Figure 6). Water-irrigated controls (0, 0 + AS04, 0 + Form) remained vigorous with 0% mortality throughout the experiment, confirming that the application of the bacterial films (0 + AS04) or the bacteria-free polymeric matrix (0 + Form) had no adverse effects on plant health compared to the untreated controls.

### 3.8. Plant Growth and Physiological

#### 3.8.1. Stem Length

**Figure 7:**
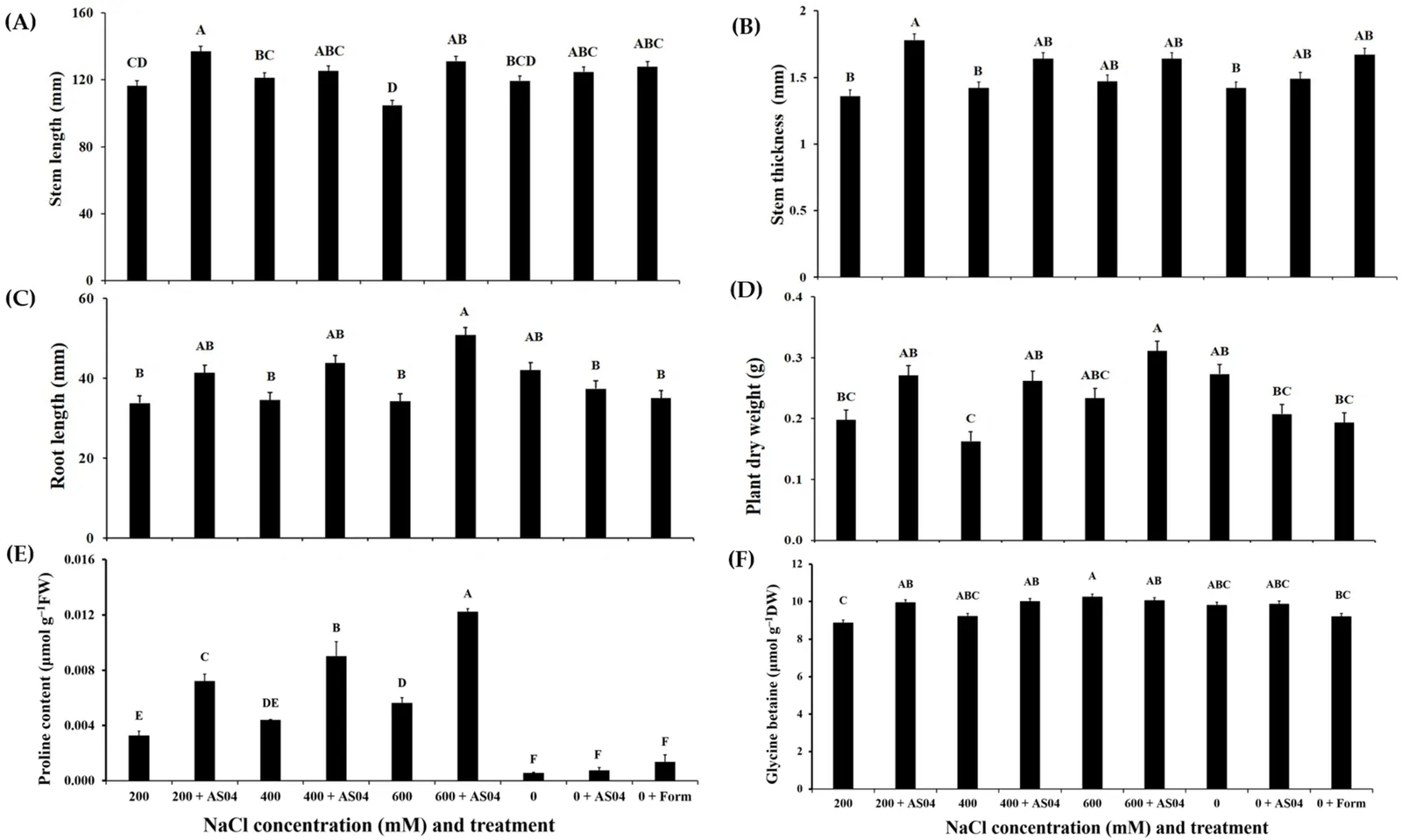
Growth and osmoprotectant responses of *Capsicum annuum* var. *glabriusculum* inoculated with *K. sediminis* AS04 under salt stress. (**A**) Stem length, (**B**) stem thickness, (**C**) root length, (**D**) plant dry weight, (**E**) proline content, and (**F**) glycine betaine content of plants exposed to 0, 200, 400, and 600 mM NaCl in the absence or presence of *K. sediminis* AS04 (+AS04). Plants treated with an immobilized bacterial formulation (Form) under non-saline conditions (0 mM NaCl) were included as additional control. Values are means ± SD. Different letters indicate significant differences among treatments (*p* < 0.05). The Kruskal–Wallis test was used to analyze glycine betaine.

Salt stress progressively reduced stem length in non-inoculated plants, with the most severe reduction observed at 600 mM NaCl (104.8 mm). Application of *K. sediminis* AS04 films significantly mitigated this effect, particularly at 200 mM, where inoculated plants achieved the greatest stem length (137.0 mm), which was not statistically different from that of water-irrigated controls (Figure 7A). At higher salinity levels (400 and 600 mM), bacterial inoculation maintained stem lengths between 125.3 and 131.1 mm, which was significantly higher than their respective non-inoculated counterparts, representing an approximate 25% increase in stem length at 600 mM NaCl compared with non-inoculated plants. Water-irrigated controls showed no growth inhibition regardless of film application.

The promotion of stem elongation by *K. sediminis* AS04 under saline conditions is consistent with responses reported in other pepper varieties, where microbial inoculation increased plant height by 9.5% in hot pepper grown in saline soil [61] and stem length by 14.6% in red pepper with *Methylobacterium oryzae* under 150 mM NaCl [62]. Similar trends have been documented in other species, including beans, where bacterial inoculation increased shoot length by 60% under salt stress [63]. This recurrent response across plant species has been attributed to plant growth-promoting mechanisms, including IAA synthesis, regulation of gibberellins and cytokinins, and ethylene reduction through ACC deaminase activity [64].

#### 3.8.2. Stem Thickness

Stem thickness varied significantly among treatments, with non-inoculated plants at 200 mM showing the lowest values (1.36 mm). Application of *K. sediminis* AS04 films significantly increased stem diameter across all salinity levels, with the greatest improvement observed at 200 mM (1.78 mm), which was statistically comparable to that of water-irrigated controls with bacterial or polymeric films (Figure 7B). At 400 and 600 mM, bacterial inoculation resulted in stem thicknesses of 1.64–1.66 mm, significantly higher than those of the respective non-inoculated counterparts. Water-irrigated controls exhibited intermediate-to-high stem thickness across treatments, indicating that bacterial films helped preserve stem structural development under salt stress.

This response is consistent with findings in *Capsicum*, where bacterial inoculation increased stem diameter by 13.68% under greenhouse conditions and up to 21.92% under water stress [65], and was observed increases of 9.9 to 27.8% in seedlings inoculated with different Bacillus strains [66]. Stem thickening has been attributed to IAA-mediated stimulation of cambial cell division, which strengthens vascular architecture and may improve water and nutrient conductance under osmotic stress [67].

#### 3.8.3. Root Morphology

Root length responded strongly to both salinity and bacterial inoculation. Non-inoculated plants under salt stress exhibited restricted root development, with average lengths of approximately 34–35 mm across all sodium chloride concentrations (Figure 7C). Application of *K. sediminis* AS04 films consistently promoted root elongation across all salinity levels, increasing root length by about 48% at 600 mM (50.6 mm), which was significantly longer than any non-inoculated treatment and was statistically like water-irrigated controls. At 200 and 400 mM, bacterial inoculation increased root length to approximately 41–44 mm. Water-irrigated controls exhibited root lengths ranging from 35 to 42 mm, indicating that bacterial inoculation effectively sustained root elongation even under severe salt stress. In contrast, root thickness (overall width of the root system) showed no significant differences among treatments, with all values ranging from 13.96 to 16.36 mm, suggesting that *K. sediminis* AS04 preferentially promotes root elongation over radial thickening under saline conditions.

The greater root promotion observed at higher salinity levels suggests that *K. sediminis* AS04 becomes increasingly relevant as stress intensifies. A similar response has been reported in *Capsicum*, where plant growth-promoting bacteria increased root length at NaCl concentrations of 100–150 mM [68,69]. This effect has been associated with IAA production, a phytohormone that plays a key role in root elongation and cell division. This trait has been documented in *K. sediminis* AC3, where auxin synthesis was directly linked to improved root development [70], and has been confirmed across multiple *Kocuria* strains, including members of the same species as this study [13]. Further evidence within the genus includes an approximately 50% increase in maize root length under 200 mM NaCl with *K. rhizophila* Y1 [71], suggesting that IAA production may similarly contribute to the root promotion observed in chiltepin with AS04.

The preferential promotion of root elongation over radial thickening suggests that *K. sediminis* AS04 directs its growth-promoting effect toward increasing absorptive surface area rather than structural root development, which may represent a more efficient adaptive strategy under osmotic stress conditions. Root elongation has been consistently reported as a key growth-promoting trait within the *Kocuria* genus under saline stress, with *K. arsenatis* ST19 promoting significant root length increases in tomato [14], and *K. turfanensis* 2M4 showing root length gains of 19–26% that outpaced root biomass increases of 12–15% under saline soil conditions [72], showing the relevance of this genus.

#### 3.8.4. Shoot Dry Biomass

Plant dry weight varied significantly among treatments (Figure 7D). Non-inoculated plants under salt stress showed reduced biomass accumulation, with the lowest value observed at 400 mM NaCl (0.16 g plant^−1^). Inoculation with *K. sediminis* AS04 increased shoot biomass across all salinity levels, raising shoot dry weight at 400 mM NaCl from 0.16 to 0.26 g plant^−1^, which represents an increase of about 61% compared with non-inoculated plants, and reaching 0.31 g plant^−1^ at 600 mM, which was statistically like water-irrigated controls and notably higher than non-inoculated plants at the same concentration.

This pattern, where bacterial inoculation becomes more effective as salinity increases, has been documented within the genus. Li et al. [71] reported that *K. rhizophila* Y1 increased shoot dry biomass by 24.7% and 50.5% under 100 and 200 mM NaCl, respectively, in maize, suggesting that enhanced biomass accumulation under saline conditions may be a shared trait in *Kocuria*. The mechanisms underlying this response may involve IAA production, which stimulates cell division and expansion in aerial tissues and has been documented across multiple *Kocuria* strains, including *K. sediminis* AC3 [70], as well as ACC deaminase activity, which reduces ethylene accumulation and relieves growth inhibition under stress, reported in the closely related *K. arsenatis* ST19 [14]. However, these traits were not directly measured in *K. sediminis* AS04 in the present study, and therefore this mechanistic explanation should be considered a working hypothesis supported by evidence from related strains, although the evidence across the genus suggests that similar mechanisms may contribute to the biomass observed in chiltepin.

#### 3.8.5. Proline Determination

Proline content increased progressively with salinity across all treatments, consistent with its established role as a compatible osmolyte that lowers cytosolic osmotic potential, thereby maintaining cellular turgor and water uptake under ionic stress. Non-inoculated plants accumulated proline from 3.26 to 4.40 µmol g^−1^ FW between 200 and 600 mM NaCl, suggesting that *Capsicum annuum* var. *glabriusculum* activates osmotic adjustment mechanisms in a dose-dependent manner under salt stress (Figure 7E). Water-irrigated controls remained low, between 0.56 and 1.37 µmol g^−1^ FW, indicating that this accumulation is a specific stress response rather than a constitutive phenotype. Inoculation with *K. sediminis* AS04 consistently increased proline content at all salinity levels, reaching 7.22, 9.03, and 11.25 µmol g^−1^ FW at 200, 400, and 600 mM NaCl, respectively, supporting a systematic, dose-dependent enhancement of osmotic adjustment. This pattern is consistent with observations in other salt-tolerant crops such as wild rice, where proline levels of about 4–5 µmol g^−1^ FW have been reported under 150 mM NaCl [73], comparable to those observed here in non-inoculated chiltepin despite the higher salinity range applied (200–600 mM NaCl. Ha-tran et al. [74], further showed that halotolerant PGPB amplifies proline production in proportion to increasing salt concentration and that, under severe osmotic stress, proline can account for up to 80% of the total free amino acid pool in inoculated plants. Species-level precedent in *Capsicum* is provided by Wang et al. [69], who demonstrated that *Bacillus* strains promoted *C. annuum* seedling growth under NaCl stress through proline-based osmotic adjustment.

Abdelkhalik et al. [63] reported increased proline content in hot pepper under saline conditions following treatment with effective microorganisms, formulated as a mixed consortium of photosynthetic bacteria (*Rhodobacter sphaeroides*, *Rhodopseudomonas palustris*), lactic acid bacteria (*Lactobacillus casei*, *Lactobacillus plantarum*, *Streptococcus lactis*), yeasts (*Candida utilis*, *Saccharomyces cerevisiae*), fermenting fungi (*Penicillium* sp., *Aspergillus oryzae*, *Mucor hiemalis*), and actinobacteria (*Streptomyces griseus*, *Streptomyces albus*). The magnitude of proline enhancement observed here, exceeding 100% relative to non-inoculated plants at 600 mM NaCl, is higher than the increments typically reported in those studies, indicating a particularly strong proline response in chiltepin inoculated with *K. sediminis* AS04 under severe salt stress.

#### 3.8.6. Glycine Betaine Determination

Glycine betaine content remained stable across all treatments, with mean values ranging between 9.6 and 10.9 μmol g^−1^ DW under saline conditions and 10.2–10.8 μmol g^−1^ DW in non-saline controls, showing no significant response to increasing NaCl concentrations or to *K. sediminis* AS04 inoculation (Figure 7F). This contrasts with the marked, dose-dependent proline accumulation documented under the same conditions, indicating that proline served as the primary osmolyte in response to both salt stress and bacterial inoculation in chiltepin. This outcome is consistent with reports indicating that endogenous glycine betaine responses to abiotic stress depend strongly on both the crop species and the genotype. In wheat (*Triticum aestivum*) and barley (*Hordeum vulgare*), increases in glycine betaine under NaCl stress were detected mainly in young leaves of plants at 18–26 days of age grown under 100–200 mM NaCl and were associated with the induction of choline monooxygenase and betaine aldehyde dehydrogenase [75]. In white clover (*Trifolium repens*), glycine betaine increased under 300 mM NaCl only in genotypes possessing a functional betaine aldehyde dehydrogenase gene, while a genotype lacking this gene showed no significant change in glycine betaine content under the same conditions [76]. In pepper (*Capsicum annuum*) seedlings exposed to low temperature combined with low light, leaves likewise did not accumulate endogenous glycine betaine in response to stress, and higher glycine betaine levels were only detected after exogenous foliar application, in agreement with previous studies on this species [77].

Although *Capsicum annuum* can accumulate both osmolytes simultaneously under salt stress, as Jin et al. [78] demonstrated in pepper seedlings, where hormonal elicitation under NaCl stress promoted significant increases in glycine betaine, which contributed to antioxidant defense and reduction of oxidative damage alongside proline and sugars. However, other authors have reported that *Capsicum annuum* does not accumulate glycine betaine under stress conditions such as salinity or drought; therefore, chili pepper is commonly reported to prefer the proline pathway as an osmoprotectant [79,80]. The differential response observed here is consistent with evidence that PGPB modulate proline and glycine betaine independently, depending on the bacterial strain and plant species [80]. Also, the exposure time may be too short for plants to activate all signaling pathways in the glycine betaine pathway. Some authors have reported similar glycine betaine concentrations (4.4–16.9 µmol g^−1^ FW) in different plant species subjected to abiotic stress for 18–20 days [81,82]. However, these values were determined using fresh tissue, which may account for differences in the reported concentrations compared with studies based on dry tissue.

Overall, the results indicate that *K. sediminis* AS04 enhances proline accumulation under salinity, while glycine betaine levels remain unchanged in chiltepin under the conditions evaluated. Given that antioxidant enzymes, oxidative damage markers, and key components of glycine betaine biosynthesis were not measured, the mechanisms underlying proline accumulation and glycine betaine stability in this system remain unresolved and warrant future investigation.

### 3.9. Multivariate Analysis of Plant Responses to Salinity and Bacterial Inoculation

**Figure 8:**
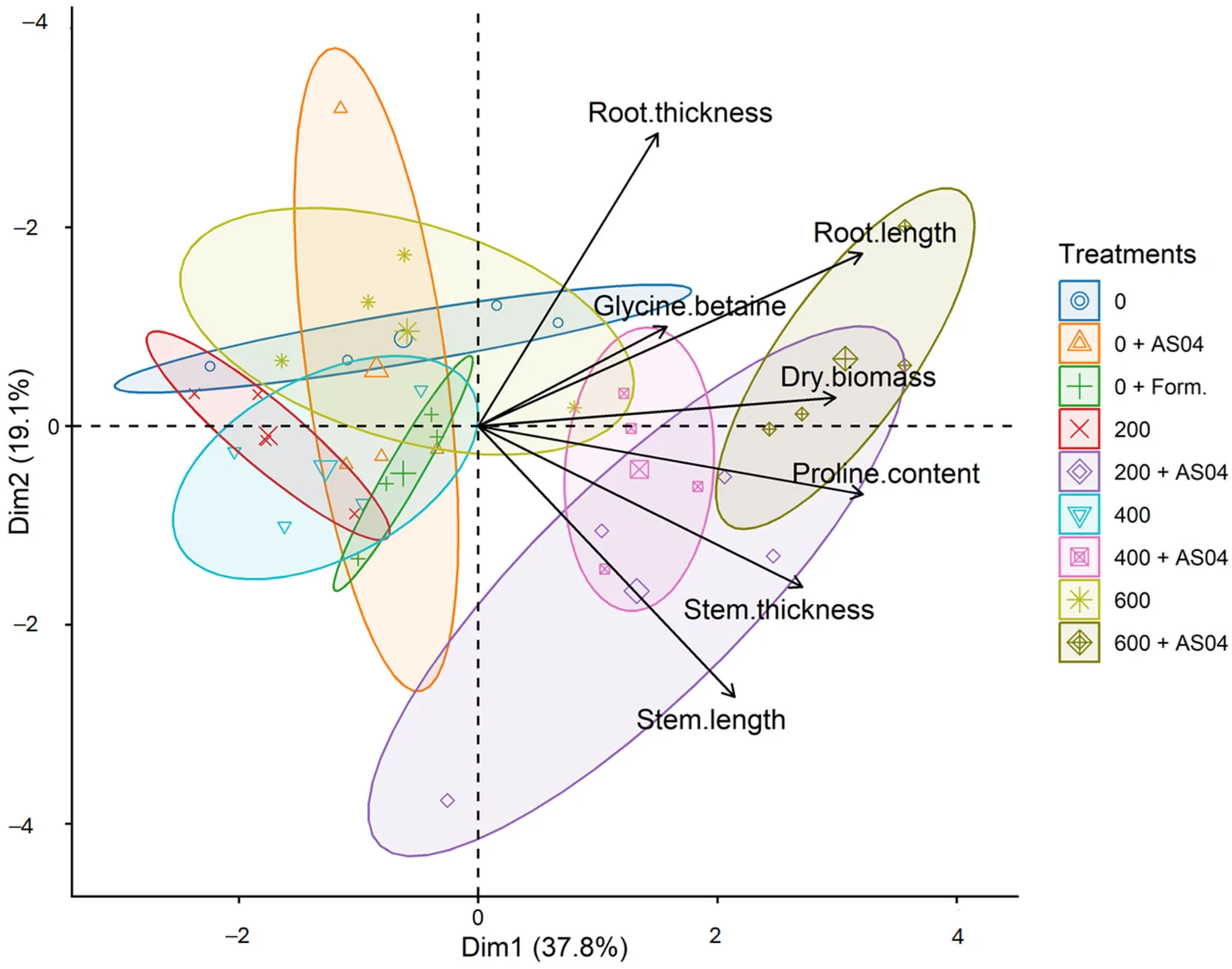
Principal component analysis of growth and physiological traits of *Capsicum annuum* var. *glabriusculum* under different salinity and *K. sediminis* AS04 inoculation treatments. Plants were exposed to 0, 200, 400, and 600 mM NaCl in the absence or presence of *K. sediminis* AS04 (+AS04). Plants treated with an immobilized bacterial formulation (Form) under non-saline conditions (0 mM NaCl) were included as additional control.

Principal component analysis of all measured plant parameters (Figure 8) showed two components explaining 56.9% of total variance (Dim1: 37.8%; Dim2: 19.1%), with the biplot revealing a clear multivariate separation among treatments driven by salinity and bacterial inoculation. Dim1, on its positive side, was grouped with proline content (0.769), root length (0.770), dry biomass (0.721), stem thickness (0.638) and stem length (0.488), which were correlated with treatments of *K. sediminis* AS04 in saline conditions, where those parameters were higher than in non-inoculated treatments. On the other hand, glycine betaine (0.394), showed limited discriminatory power, reflecting its lack of significant variation among treatments. Although retained in the PCA due to its physiological relevance in osmotic adjustment, this variable was not interpreted as a major factor driving treatment separation. In contrast, the negative side of Dim1 was associated with treatments showing comparatively lower values for these stress-related traits, reinforcing that this axis captures a salt-tolerance gradient linked to bacterial inoculation. The clustering of proline, root length, dry biomass, stem thickness, and stem length on the same side of Dim1 indicates that these variables correlated positively across treatments. This pattern suggests that enhanced osmotic adjustment, particularly through proline accumulation, was closely associated with sustained root development and preservation of aerial growth under saline conditions.

The highest proline loading is consistent with its role as the main osmolyte activated under salt stress and enhanced by *K. sediminis* AS04. Overall, Dim1 can therefore be interpreted as an integrated tolerance axis that summarizes the positive association between proline accumulation and the preservation of growth-related traits in inoculated plants.

Dim2 reveals a contrast in treatments based on root and stem traits: root thickness (overall width of the root system) (0.681) and root length (0.396) load upward, while stem length (−0.678) and stem thickness (−0.425) load downward. This pattern indicates an opposing gradient between enhanced root development and increased aerial growth, largely independent of the salinity-inoculation pattern shown in Dim1. The shift of the 0 + AS04 treatment toward the upper-left quadrant suggests that bacterial inoculation under non-saline conditions results in a distinct morphological profile marked by thicker roots and altered shoot architecture, implying a growth-promoting effect of *K. sediminis* AS04 in control conditions. Glycine betaine shows moderate correlation in Dim1 (0.394) and Dim2 (0.211), indicating a limited role in separating treatments in the two-dimensional analysis, which aligns with its relatively low variation in the main PCA plane. Its lower loading compared to proline supports the idea that proline, not glycine betaine, is more closely associated with the multivariate stress-tolerance profile of inoculated chiltepin plants.

PCA integrated the individually discussed parameters into a coherent picture in which bacterial inoculation shifted the overall plant phenotype toward a coordinated stress-tolerance response, where proline accumulation, sustained root elongation, and biomass preservation appeared as closely associated traits rather than independent responses [81].

The use of agave leaf juice, which is typically a waste in the agave-tequila chain, could be an opportunity to revalue it and improve the circular economy in this sector. It is a nutrient source for microbial biomass production, such as PGPB, which can be applied to crops to protect against salinity and improve plant growth, making it a sustainable tool in the agricultural sector. However, Future studies should investigate the molecular mechanisms underlying plant–microbe interactions and salt stress alleviation.

## 4. Conclusions

This study demonstrates that *Agave tequilana* leaf juice supplemented with urea is a viable alternative to conventional tryptone-based media for *Kocuria sediminis* AS04 biomass production, sustaining high cell densities and reproducible viable cell concentrations suitable for formulation and delivered via biopolymeric film inoculants provided clear benefits to *Capsicum annuum* var. *glabriusculum* under severe salt stress, reflected in higher survival, preserved shoot growth and biomass, consistent root elongation promotion, and marked proline accumulation, suggesting that this strain helps maintain plant growth and osmotic adjustment under salt stress. Nevertheless, the present study was conducted under controlled growth chamber conditions, and validation under field or greenhouse conditions will be necessary to confirm the performance of *K. sediminis* AS04 biopolymeric film inoculants in salt-affected agricultural systems. Future work should evaluate rhizosphere colonization by *K. sediminis* AS04, its long-term performance under field-relevant conditions, and its molecular mechanisms. By integrating an *agave* leaf-based medium for bacterial production and subsequently applying it to chiltepin under salinity stress, this work addresses both perspectives: the high costs of PGPB inoculant production and the valorization of agroindustrial waste. Using *agave* leaf juice at 25% (*v*/*v*) of the culture medium enables proportional water savings at an industrial scale, reinforcing the environmental relevance of this formulation. Further testing under commercial-scale conditions is recommended. This integrated approach provides a cost-effective and sustainable strategy for chiltepin production in salt-affected agricultural systems, aligning with circular bioeconomy principles by valorizing agave leaf residues.

## Figures and Tables

**Table 1:** Code experimental treatments, NaCl concentrations and type of inoculation applied to *C. annuum* var. *glabriusculum* plants.

Code	NaCl in Irrigation (mM)	Inoculation
200	200	Non-inoculated
200 + AS04	200	*K. sediminis* AS04 films
400	400	Non-inoculated
400 + AS04	400	*K. sediminis* AS04 films
600	600	Non-inoculated
600 + AS04	600	*K. sediminis* AS04 films
0	0	Non-inoculated
0 +AS04	0	*K. sediminis* AS04 films
0 + Form *	0	Non-inoculated

* Form: biopolymeric film formulation without bacteria (matrix control). All treatments consisted of *n* = 10 replicates in a completely randomized design.

**Table 2:** Characterization of *agave* leaf juice.

Parameter	This Study	Literature Values	References
pH	5.14 ± 0.01	5.29 ± 0.05	[35]
Electrical conductivity (mS cm^−1^)	10.95 ± 0.08	-	-
Salinity (%)	0.47 ± 0.15	-	-
Total carbohydrates (g L^−1^)	24.83 ± 4.43	23.3–35	[21]
Fructose (g L^−1^)	12.79 ± 0.01	26.6	[36]
Glucose (g L^−1^)	5.26 ± 0.03	7.0	[37]
Sucrose (g L^−1^)	1.49 ± 0.01	5.9	[38]
Inulin (g L^−1^)	47.90 ± 0.18	38.41–43.86	[39]
Phenols (g GAE L^−1^) *	1.27 ± 0.03	2.91	[40]
Total organic carbon TOC (g L^−1^)	27.44 ± 0.33	32.03 ± 1.56	[41]
Total phosphorus (g L^−1^)	1.21 ± 0.04	0.087	[41]
Total nitrogen (g L^−1^)	0.22 ± 0.01	0.087–0.095	[42]

* Gallic acid equivalents (GAE).

**Table 3:** Growth of *K. sediminis* AS04 on TSA supplemented with different percentages of *A. tequilana* leaves juice, and total carbohydrates in the juice.

*Agave* Juice (% *v*/*v*)	Total Carbohydrates (g L^−1^)	*K. sediminis* (CFU mL^−1^)
(TSA control)	-	(1.06 ± 0.98) × 10^8 a^
25	6.20	(1.19 ± 1.01) × 10^8 a^
50	12.4	(1.07 ± 2.38) × 10^8 a^
100	24.8	ND ^b^ *

* ND = Not detected. Different letters indicate significant differences among treatments. Kruskal–Wallis/Dunn, α = 0.05.

## Data Availability

The original contributions presented in this study are included in the article and Supplementary Materials. Further inquiries can be directed to the corresponding authors.

## Supplementary Materials

The following supporting information can be downloaded at: https://www.mdpi.com/article/10.3390/biotech15030067/s1, Figure S1: Optical density and viable cell count of *Kocuria sediminis* in TSB with different agave juice concentrations. Figure S2: Optical density of *Kocuria sediminis* in agave juice with different nitrogen sources and TSB control. Figure S3: Stability of immobilized *Kocuria sediminis* AS04 during accelerated storage. Figure S4: Chiltepin pepper plants under salt stress with or without *Kocuria sediminis* AS04. Figure S5: Substrate electrical conductivity after saline treatments. Table S1: Survival of *Capsicum annuum* var. *glabriusculum* seedlings exposed to 200, 400, and 600 mM NaCl with or without *Kocuria sediminis* AS04 inoculation (surviving/total plants [%]).
