# Supplementary material for: Valorization of Agave Leaf Juice for Optimized Kocuria sediminis AS04 Production and Its Delivery via Immobilized Films to Mitigate Saline Stress in Capsicum annuum var. glabriusculum
Source: BioTech (Basel). 2026 Aug 15;15(3):67. doi: 10.3390/biotech15030067 (PMC13510538; doi:10.3390/biotech15030067)
Supplement: Supplementary file 1 [file biotech-15-00067-s001.zip › biotech-4371489-supplementary.pdf]

BioTech

MDPI

Supplementary Materials: Valorization of *Agave* Leaf Juice for Optimized *Kocuria sediminis* AS04 Production and Its Delivery via Immobilized Films to Mitigate Saline Stress in *Capsicum annuum var. glabriusculum*

Claudia Estefania Cabrera-Muro, Rosa María Camacho-Ruiz, Miguel Angel Lorenzo-Santiago, Jacobo Rodriguez-Campos and Silvia Maribel Contreras-Ramos

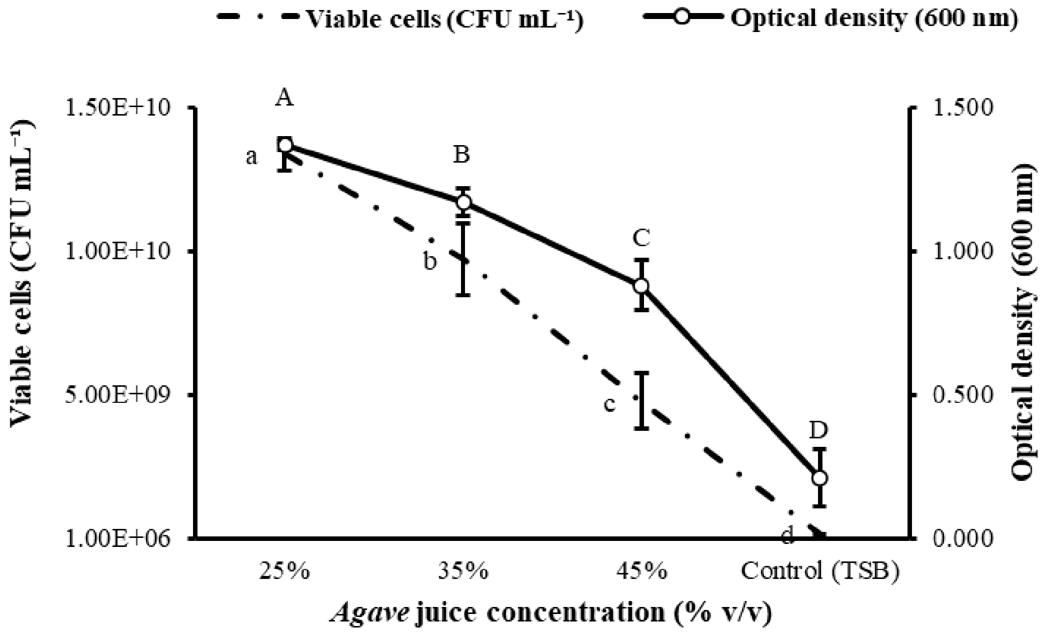

Figure S1. Viable cell counts (CFU mL^{-1}) and optical density at 600 nm (OD_{600}) of *Kocuria sediminis* grown in tryptic soy broth (TSB) supplemented with 25%, 35%, or 45% (v/v) agave juice, and in unsupplemented TSB (control). Dashed and solid lines represent CFU mL^{-1} and OD_{600}, respectively; data points are means ± SD (n = 3). Different capital and lowercase letters indicate significant differences in OD_{600} and CFU mL^{-1}, respectively (Tukey's test, $p < 0.05$).

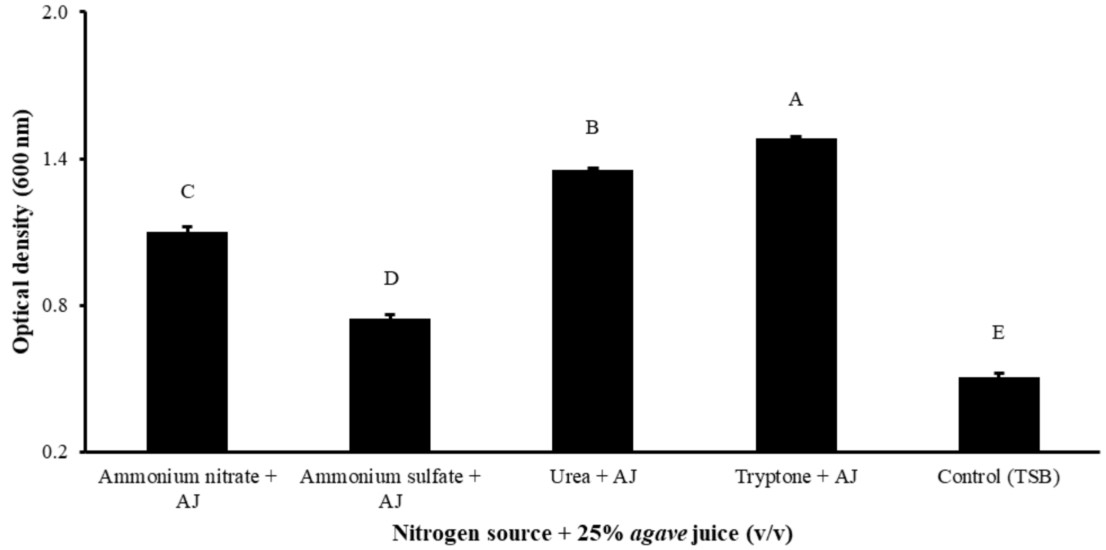

Figure S2. Optical density at 600 nm (OD_{600}) of *Kocuria sediminis* AS04 grown in 25% (v/v) agave juice (AJ) supplemented with different nitrogen sources and TSA (control). Bars represent ± SD (n = 3). Different capital letters indicate significant differences among treatments (Tukey's test, $p < 0.05$).

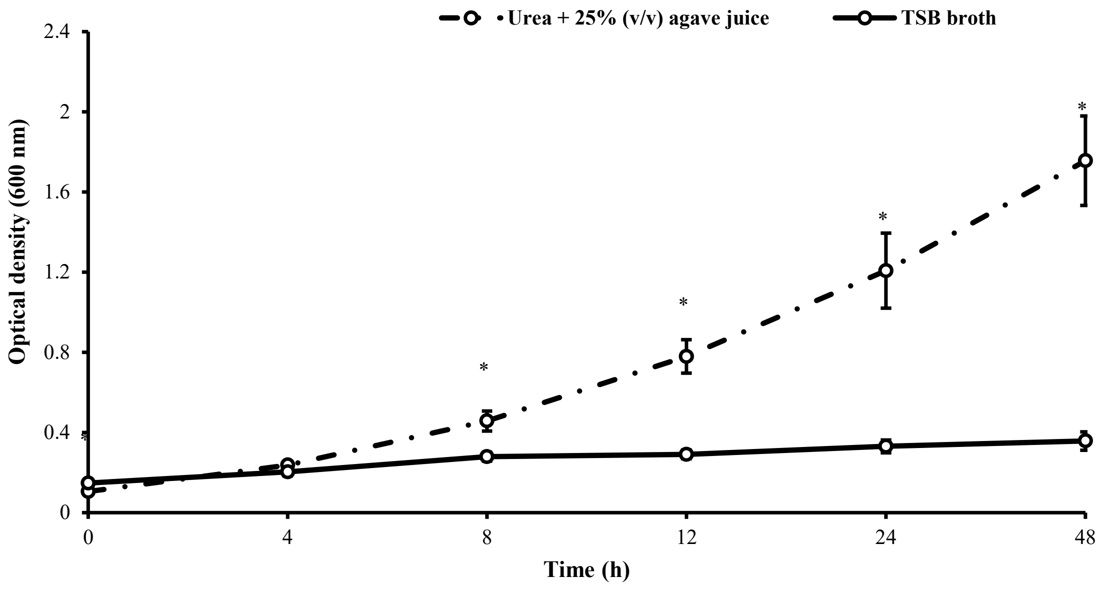

Figure S3. Optical density at 600 nm (OD_{600}) of *Kocuria sediminis* AS04 grown over 48 h in TSB or in 25% (v/v) agave juice supplemented with urea. Data points are means ± SD (n = 3). Asterisks indicate significant differences between media at each time point (Welch's t-test, $p < 0.05$).

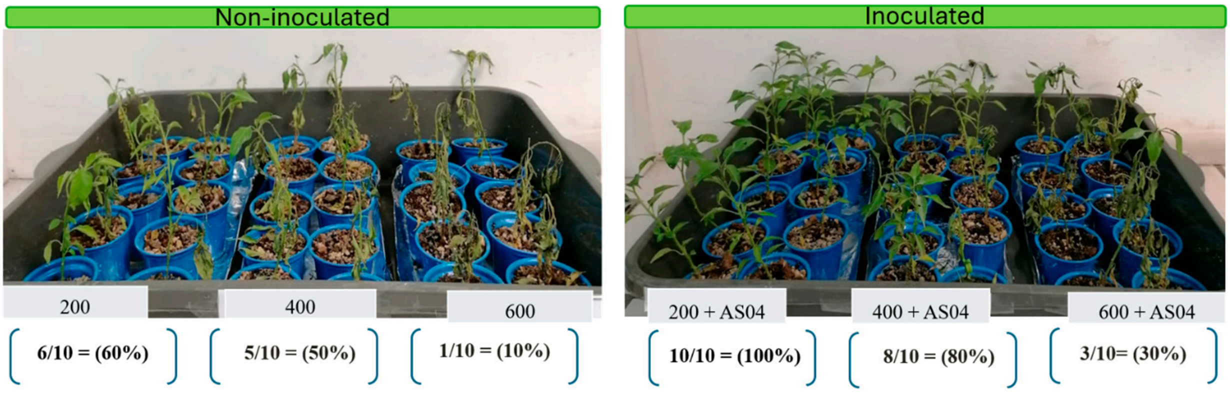

Figure S4. Appearance and survival of *Capsicum annuum* var. *glabriusculum* seedlings at the end of the salt stress experiment. Left and right panels show non-inoculated and inoculated plants (*Kocuria sediminis* AS04), respectively, at 200, 400, and 600 mM NaCl. Labels indicate the number of surviving seedlings out of the total and the survival percentage per treatment.

Table S1. Survival of *Capsicum annuum* var. *glabriusculum* seedlings exposed to 200, 400, and 600 mM NaCl with or without *Kocuria sediminis* AS04 inoculation (surviving/total plants [%]).

NaCl (Mm)	Non- inuculated	Inoculated with *k. sediminis* AS04
200	60%	100%
400	50%	80%
600	10%	30%

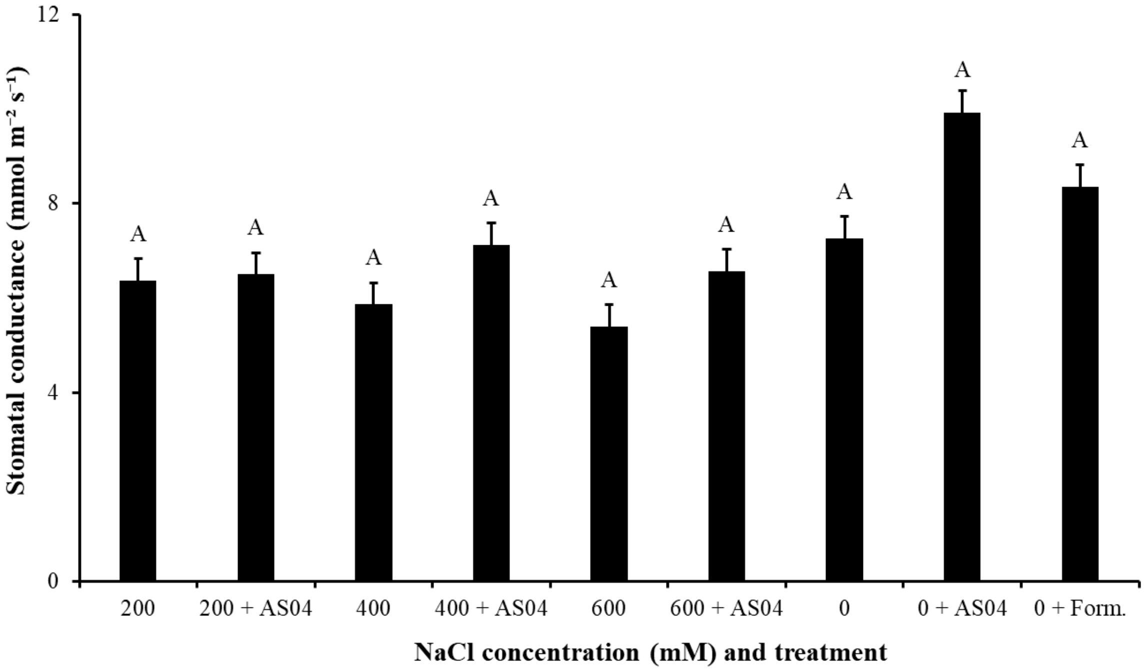

Figure S5. Stomatal conductance (g_s, mmol m^{-2} s^{-1}) of *Capsicum annuum* var. *glabriusculum* (chiltepín) under 200, 400, and 600 mM NaCl, with or without *Kocuria sediminis* AS04 inoculation, and water-irrigated controls. No significant differences were detected among treatments (Kruskal–Wallis, p = 0.178).
